# Recent Advances in Hydrogel-Promoted Photoelectrochemical Sensors

**DOI:** 10.3390/bios15080524

**Published:** 2025-08-10

**Authors:** Yali Cui, Yanyuan Zhang, Lin Wang, Yuanqiang Hao

**Affiliations:** 1 College of Chemistry and Chemical Engineering, Zhoukou Normal University, Zhoukou 466001, China; 20221021@zknu.edu.cn (Y.C.); zyy1212001@163.com (Y.Z.); 2 Department of Ophthalmology, Hunan Aerospace Hospital, Hunan Normal University, Changsha 410081, China; 3 Key Laboratory of Theoretical Organic Chemistry and Functional Molecule of Ministry of Education, School of Chemistry and Chemical Engineering, Hunan University of Science and Technology, Xiangtan 411201, China

**Keywords:** photoelectrochemical sensors, hydrogel, detection

## Abstract

Photoelectrochemical (PEC) sensors have garnered increasing attention due to their high sensitivity, low background signal, and rapid response. The incorporation of hydrogels into PEC platforms has significantly expanded their analytical capabilities by introducing features such as biocompatibility, tunable porosity, antifouling behavior, and mechanical flexibility. This review systematically categorizes hydrogel materials into four main types—nucleic acid-based, synthetic polymer, natural polymer, and carbon-based—and summarizes their functional roles in PEC sensors, including structural support, responsive amplification, antifouling interface construction, flexible electrolyte integration, and visual signal output. Representative applications are highlighted, ranging from the detection of ions, small biomolecules, and biomacromolecules to environmental pollutants, photodetectors, and flexible bioelectronic devices. Finally, key challenges—such as improving fabrication scalability, enhancing operational stability, integrating emerging photoactive materials, and advancing bio-inspired system design—are discussed to guide the future development of hydrogel-enhanced PEC sensing technologies.

## 1. Introduction

Photoelectrochemical (PEC) sensing integrates the optical excitation of photoactive materials with electrochemical signal transduction, offering a unique dual-modality detection mechanism that distinguishes it from traditional electrochemical and spectroscopic methods [1,2,3,4,5,6,7,8,9,10,11,12]. By converting light-induced charge separation events into measurable photocurrent or photovoltage outputs, PEC sensors enable highly sensitive detection with low background noise, simplified instrumentation, and excellent miniaturization potential. Unlike conventional electrochemical techniques that rely solely on electrical stimulation, PEC systems utilize light as the excitation source, which reduces interference and enhances signal resolution, especially in complex biological or environmental matrices. Over the past decade, significant progress has been made in engineering PEC-active materials—including inorganic semiconductors [13,14,15,16,17], like metal oxides [18,19,20,21,22,23,24], metal sulfides [25,26,27,28,29], and heterojunction composites [30,31,32,33,34], as well as organic photoactive species, such as graphitic carbon nitride [35,36,37,38,39,40], conducting polymers [41,42,43,44], and small organic photosensitizers [45,46,47,48,49,50,51,52,53,54,55,56,57]. These developments have fueled the rapid evolution of PEC sensing platforms, enabling the detection of a broad range of analytes, from ions [58,59,60] and small molecules [61,62,63,64,65,66,67,68,69,70,71,72] to biomacromolecules [73,74,75,76,77,78] and cells [79,80,81,82,83]. Notably, recent innovations have also expanded the boundaries of PEC biosensing through the integration of nanostructured materials, multi-functional receptors, and signal amplification strategies, further improving analytical performance in terms of sensitivity, selectivity, and real-sample applicability. Nevertheless, despite these notable advances, PEC sensing systems still face several critical challenges that hinder their widespread application, especially in the field of bioanalysis. The performance of PEC sensors is highly dependent on the stability and compatibility of photoactive materials, which are often prone to photodegradation, lack long-term durability, and exhibit poor biocompatibility. In complex sample matrices, signal fluctuation caused by nonspecific interference further compromises detection reliability. Moreover, the seamless integration of light-harvesting units, recognition elements, and transduction layers within a compact architecture remains technically challenging, particularly for flexible or portable devices. These limitations highlight an urgent need for adaptive interfacial materials capable of supporting structural flexibility, multifunctional integration, and operational stability under dynamic conditions.

Hydrogels are a class of hydrophilic polymeric networks that can absorb and retain significant amounts of water while maintaining a solid-like framework [84,85,86]. Their high-water content, soft yet elastic texture, and tunable physicochemical properties have made them increasingly attractive in a wide array of sensing platforms [87,88,89]. Depending on their synthesis strategy, hydrogels can be physically or chemically cross-linked, leading to differences in mechanical strength, porosity, and reversibility. These characteristics allow hydrogels to be engineered with tailored responses to a variety of environmental stimuli, such as pH [90,91,92], temperature [93,94,95], ionic strength [96,97,98], and biochemical signals [99,100,101]. In the context of chemical sensing and biosensing, hydrogels offer several unique advantages. First, their three-dimensional porous architecture facilitates efficient mass transport of analytes, enabling rapid interaction with embedded recognition elements. Second, their soft and hydrated nature ensures biocompatibility and allows the encapsulation of delicate biomolecules, such as enzymes, aptamers, nucleic acids, or even whole cells, without compromising their bioactivity. Third, hydrogels can serve as versatile scaffolds for the incorporation of nanomaterials, redox species, or signal transducers, thus supporting signal amplification or functional integration. These features are particularly beneficial when constructing interfaces between biological systems and transducers in electrochemical and photoelectrochemical devices [102,103,104,105,106]. Moreover, emerging trends have highlighted the role of hydrogels as “smart” interfaces that dynamically modulate sensing behavior. Volume phase transitions in stimuli-responsive hydrogels can trigger changes in permeability, conductivity, or optical properties, allowing for signal gating, amplification, or logic operations. The convergence of hydrogel chemistry with nanotechnology has also enabled the development of hybrid hydrogels with enhanced mechanical strength, self-healing ability, and multifunctionality. Such advances have extended their applicability beyond in vitro diagnostics to wearable, implantable, and environmental sensing systems.

The integration of hydrogels into photoelectrochemical (PEC) sensing systems has introduced a new dimension in the design of functional interfaces that bridge soft matter with solid-state photoactive elements. Beyond serving as passive substrates or immobilization media, hydrogels can actively modulate PEC performance through their ionic conductivity, environmental responsiveness, and capacity to interface with both biological and synthetic recognition units [107,108,109,110]. Their hydrated networks can facilitate charge transport, control mass diffusion, and selectively interact with analytes, making them particularly suited for enhancing the sensitivity, specificity, and operational stability of PEC sensors. Notably, hydrogels can be rationally engineered to fulfill distinct roles depending on the demands of the sensing architecture. For example, conductive or redox-active hydrogels can participate in electron transfer processes, while stimuli-responsive gels can be designed to undergo phase transitions or release encapsulated probes upon target recognition. In certain systems, the hydrogel itself functions as a transduction layer, amplifying or attenuating photocurrents through light-modulated physical changes. These diverse functionalities allow hydrogels to contribute not only to analytical signal generation but to sample compatibility, device miniaturization and, in some cases, visual output for portable or point-of-need applications.

**Scheme 1:**
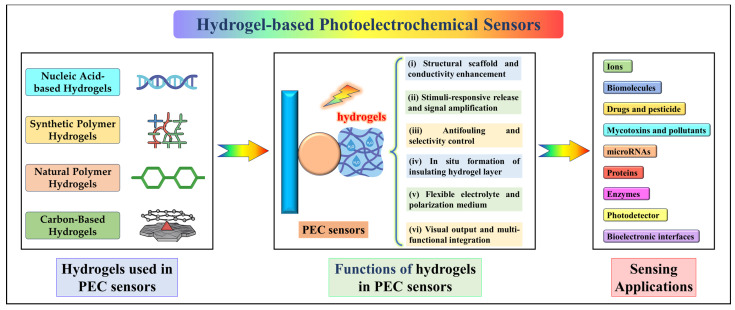
Schematic illustration of hydrogel-based photoelectrochemical (PEC) sensors, highlighting the classification of hydrogel types, their functional roles in PEC systems, and representative application scenarios.

Given the increasing number of reports highlighting hydrogel-based PEC sensing platforms, there is a clear need to consolidate and categorize the ways in which hydrogels contribute to performance improvement. This review aims to provide a comprehensive overview of recent developments in this area, focusing on the material types, structural designs, and sensing mechanisms involved (Scheme 1). We begin by categorizing hydrogel materials based on their chemical composition—such as nucleic acid-based, synthetic polymer, natural polymer, and carbon-based systems—followed by a discussion of their specific roles in PEC platforms, including conductivity enhancement, target-responsive release, antifouling, flexible electrolyte functions, and optical integration. Furthermore, we summarize the recent advances in hydrogel-assisted PEC sensors across various analytical targets, including metal ions, small biomolecules, toxins, nucleic acids, and macromolecular proteins. We also extend our discussion to emerging directions, such as hydrogel-enabled photodetectors, bioelectronic devices, and integrated imaging platforms. By mapping the intersection of hydrogel science and PEC technology, this review aims to elucidate fundamental principles, highlight current challenges, and suggest promising pathways for future research.

## 2. Hydrogel Types and Functional Roles in PEC Sensors

Before delving into the specific functions that hydrogels fulfill in photoelectrochemical (PEC) sensors, it is necessary to first understand the chemical diversity and structural classifications of these materials. The type of hydrogel used can profoundly influence its interaction with photoactive components, its responsiveness to external stimuli, and its compatibility with target analytes or biological systems. In this section, we first categorize the hydrogels commonly employed in PEC platforms based on their origin and composition, followed by a discussion of how these materials contribute functionally to the performance and versatility of PEC devices.

### 2.1. Classification of Hydrogels in PEC Sensors

**Figure 1:**
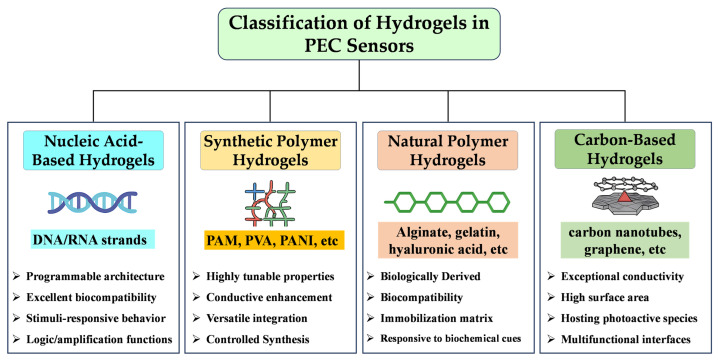
Schematic illustration of the hydrogel types used in PEC sensor.

Hydrogels employed in PEC sensors can be broadly categorized according to their chemical origin, network structure, and functional purpose. Each class offers distinct advantages in terms of mechanical properties, biocompatibility, responsiveness, and compatibility with photoactive or recognition elements. This subsection outlines four representative categories—nucleic acid-based, synthetic polymer, natural polymer, and carbon-based hydrogels—highlighting their material characteristics and the rationale behind their integration into PEC sensing systems (Figure 1). However, this classification by chemical origin does not fully reflect function-critical features, such as pore architecture, viscoelasticity, or hydration-dependent mass transport efficiency, which also merit consideration when optimizing hydrogel–sensor pairings.

#### 2.1.1. Nucleic Acid-Based Hydrogels

Nucleic acid-based hydrogels are typically constructed from DNA strands through sequence-specific hybridization, enzymatic ligation, or crosslinking via aptamer–target interactions. Their programmability and biocompatibility make them ideal for biosensing applications where molecular recognition is key. In PEC systems, DNA hydrogels are often employed as intelligent scaffolds that respond to specific targets—such as microRNAs or enzymes—through structural collapse or network degradation [111,112,113]. This target-induced transition can be coupled to the release of embedded signal reporters, such as semiconductor nanoparticles or photosensitizers, which in turn regulate the PEC output. The ability to encode logic behavior and amplification cascades within the DNA framework adds another layer of control over signal modulation in nucleic acid-based hydrogel PEC platforms. Compared to other hydrogel types, DNA hydrogels exhibit superior specificity and are especially advantageous in applications requiring programmable response and molecular logic control. However, they are generally less stable under harsh environmental conditions and may involve complex synthesis and higher costs, limiting their scalability. Recent studies have demonstrated that DNA hydrogels can also function as physical barriers to modulate electron transfer, as programmable reservoirs for enzyme encapsulation, or as homogeneous signal-generation matrices in CRISPR-mediated or enzyme-amplified PEC systems [111,113,114]. These features greatly expand their functional versatility in advanced PEC biosensors. However, their dense crosslinked networks may impose diffusion limitations, especially for high molecular-weight analytes, potentially increasing response times. Although widely used in laboratory biosensors, their performance in ionic-strength-variable real-world samples remains largely untested.

#### 2.1.2. Synthetic Polymer Hydrogels

Synthetic hydrogels, based on polymers like polyacrylamide (PAM), polyvinyl alcohol (PVA), or polyaniline (PANI), offer excellent tunability in terms of mechanical strength, electrical conductivity, and functional group incorporation. These hydrogels can serve both as structural matrices and as conductive elements in PEC sensors [107,115,116,117]. Their flexibility in synthesis allows for the incorporation of conductive polymers, redox mediators, and photoactive nanoparticles to improve electron transfer efficiency and signal output. For instance, conductive hydrogels based on PANI have been developed to serve as both electron transporters and porous scaffolds. These structures facilitate mass transport, enhance light-harvesting efficiency, and enable the stable integration of photoactive donor–acceptor materials [117]. Similarly, in situ generated PANI networks within PEC platforms have been shown to synergize with enzymatic reactions (e.g., glucose oxidase), enabling H_2_O_2_-triggered signal amplification via oxidative polymerization, thereby boosting the photocurrent response [112]. Compared with DNA hydrogels, synthetic polymer hydrogels exhibit better long-term stability, greater chemical versatility, and more scalable production. They are particularly suitable for PEC systems requiring antifouling, mechanical robustness, or conductivity modulation. However, their lack of inherent biorecognition capability may necessitate additional functionalization steps. Moreover, hydrogels based on polyacrylamide or zwitterionic copolymers have been engineered to improve antibiofouling properties in PEC sensors for in vivo applications. These systems, when combined with metal–organic frameworks (MOFs) or nanostructured semiconductors, form bioinert and photoconductive interfaces that resist nonspecific protein adsorption and maintain high sensitivity during biological detection [118]. Synthetic hydrogels often demonstrate improved chemical robustness and tunable permeability, but the lack of inherent biological specificity requires additional functionalization strategies. Furthermore, the swelling behavior of these hydrogels under physiological pH and ionic strength can alter interfacial impedance and sensor reproducibility.

#### 2.1.3. Natural Polymer Hydrogels

Hydrogels derived from natural sources—such as alginate (Alg), chitosan, hyaluronic acid, and gelatin—offer distinct advantages in terms of biocompatibility, biodegradability, and environmental safety. Their soft, hydrated networks are well suited for interfacing with biological samples or living systems. In PEC sensing, natural hydrogels often act as immobilization media for enzymes, aptamers, or nanoparticles, while simultaneously providing a permeable matrix that facilitates substrate diffusion and electron transfer [69,108,109,119]. Natural hydrogels are preferable in bioanalytical applications where biosafety and in vivo compatibility are critical. However, they typically exhibit weaker mechanical strength and lower electrical conductivity compared to synthetic or carbon-based hydrogels, which may limit their applicability in wearable or high-load PEC systems. Recent studies have demonstrated a wide range of innovative applications of natural polymer hydrogels in PEC systems. For instance, alginate hydrogel was employed to form a stimulus-responsive layer through Ca^2+^ crosslinking on Ti_3_C_2_@Bi_2_WO_6_ photoelectrodes, which significantly hindered interfacial charge transfer and light absorption, enabling ultrasensitive detection of Aβ_1–42_ via photocurrent suppression [119]. Similarly, mixed cellulose/carboxymethyl cellulose (CMC) hydrogels have been introduced as flexible solid electrolytes in PEC-type photodetectors. These cellulose-based hydrogels provided excellent mechanical flexibility and ionic conductivity, allowing for stable photoresponses over multiple bending cycles, thereby offering strong prospects for wearable optoelectronics [109]. Moreover, gelatin-based hydrogels have been integrated with semiconductors or redox polymers to construct organic PEC transistors for protein sensing [108]. These biohybrid materials not only enable efficient signal modulation but facilitate target-induced changes in optical/electrical behavior. In summary, natural polymer hydrogels serve not only as biocompatible matrices but as functional modulators in PEC systems, enabling enhanced sensitivity, flexible integration, and intelligent response capabilities.

#### 2.1.4. Carbon-Based Hydrogels

Carbon-based hydrogels, such as those derived from graphene oxide or carbon nanotubes, exhibit exceptional conductivity, mechanical robustness, and surface area. When integrated into PEC platforms, these hydrogels function as both electron-conducting networks and scaffolds for anchoring photoactive species [120,121,122,123]. Their hierarchical porosity facilitates light penetration, mass transport, and efficient charge collection, which are critical for improving PEC sensor performance. For example, 3D nitrogen-doped graphene hydrogels (NGH) have been used to support semiconductor sensitizers, such as MoS_2_ or BiPO_4_, forming p–n heterojunctions that promote effective separation of photogenerated charge carriers and enhance photocurrent response [124,125]. Carbon-based hydrogels are advantageous in PEC systems requiring high conductivity, mechanical strength, and photostability. They are particularly effective for environmental and electrochemical applications. However, they lack intrinsic biological recognition and responsiveness, requiring additional functionalization for bio-targeted sensing. Similarly, dual 3D structural Bi_2_WO_6_@graphene hydrogel (GH) composites have been integrated with molecularly imprinted polymers (MIPs) for the detection of 4-nitrophenol, where the graphene hydrogel not only facilitated charge transfer but improved light utilization and analyte adsorption [123]. In ratiometric PEC systems, carbon-based hydrogels have also been used to load carbon quantum dots (C-dots), forming C-dots/3DGH structures that produce cathodic photocurrent signals, enabling dual-signal output in combination with anodic references like g-C_3_N_4_ [126]. Moreover, the good electrocatalytic activity and flexibility of carbon hydrogels make them suitable for miniaturized and wearable PEC devices, especially for environmental pollutant detection and pathogen monitoring. The synergistic combination of carbon-based frameworks with metal oxides or conjugated polymers further broadens the functional scope of these hydrogels, enabling tunable electronic structures and multifunctional PEC interfaces.

Emerging hydrogel variants—such as self-healing hydrogels, supramolecular gels, and injectable or spray-deposited formulations—offer exciting opportunities to overcome traditional mechanical and processability constraints. Moreover, integrating hydrogel-PEC platforms with AI-based pattern recognition algorithms may advance intelligent sensing and automated decision-making.

### 2.2. Functional Roles of Hydrogels in PEC Systems

Hydrogels play an active and multifaceted role in enhancing the performance of PEC sensing systems through several interrelated mechanisms. Their hydrated, ionically conductive networks help reduce interfacial resistance and promote efficient charge transfer between the photoelectrode and the electrolyte. The porous three-dimensional architecture facilitates rapid analyte diffusion and efficient interaction with recognition elements, thereby accelerating reaction kinetics and improving sensor responsiveness. Additionally, the inherent softness and flexibility of hydrogels allow conformal contact with microstructured electrodes, minimizing charge leakage and enhancing signal stability. Importantly, the abundant functional groups (e.g., –COOH, –NH_2_, –OH) in hydrogel matrices can modulate the local interfacial microenvironment—such as pH or redox potential—around photoactive sites, thereby improving charge separation and transfer efficiency. In stimuli-responsive systems, hydrogels may undergo structural changes or trigger the release of photoactive species in response to external signals, enabling the dynamic control of signal generation. Collectively, these synergistic mechanisms underline the essential role of hydrogels as active interfacial components in PEC sensors. The following subsections elaborate on their key functions in terms of structural support, interface modulation, and performance enhancement.

#### 2.2.1. Structural Scaffold and Conductivity Enhancement

Hydrogels with three-dimensional porous networks serve as effective frameworks for anchoring photoactive materials, redox mediators, and recognition elements. Their highly hydrated, permeable structure promotes analyte diffusion and enhances the contact between the light-absorbing material and the electrolyte [116,123]. When doped with conductive polymers (e.g., polyaniline or PEDOT) or integrated with carbon-based nanomaterials, such hydrogels can also assist in charge transport across the sensing interface [117,127]. For instance, conductive hydrogels incorporating donor–acceptor-type polymers and PANI networks have been used to build highly sensitive PEC biosensors, offering both electrical continuity and favorable porosity for light penetration and mass transport [117]. In other systems, ferrocene (Fc)-grafted polyethylene glycol (PEG) hydrogels anchored onto silicon photoelectrodes provided stable, light-switchable electron channels, enabling reproducible and multiplexed enzymatic biosensing under ambient conditions [128]. Moreover, the mechanical softness and adaptability of hydrogel frameworks make them especially suitable for interfacing with miniaturized, deformable, or wearable PEC devices. Their structural and conductive dual function plays a key role in enhancing sensitivity, operational stability, and integration flexibility in advanced PEC sensors.

#### 2.2.2. Stimuli-Responsive Release and Signal Amplification

Smart hydrogel matrices can be programmed to undergo structural transitions or controlled degradation in response to external triggers, such as target binding, enzymatic activity, or environmental changes. These transformations are often coupled with the release of encapsulated photoactive species (e.g., quantum dots, dyes, or redox agents), which amplify the PEC signal upon liberation [110,112,114,119]. For instance, hyaluronic acid (HA)-based hydrogels have been utilized to encapsulate crystal violet (CV), a visible-light photosensitizer. Upon exposure to hyaluronidase (HAase), the enzyme specifically degrades the HA network, releasing CV to sensitize BiOBr nanoflowers and thereby enhancing the cathodic photocurrent response in a “signal-on” PEC format [110]. Similarly, DNA hydrogels have been engineered to respond to nucleic acid targets or CRISPR/Cas systems. A CRISPR/Cas12a-responsive DNA hydrogel was used to regulate the formation of photoactive heterojunctions. Target-triggered rolling circle amplification (RCA) activated Cas12a, which nonspecifically cleaved ssDNA cross-links in the DNA hydrogel. This degradation allowed azide-modified CdS quantum dots to react with DBCO-functionalized g-C_3_N_4_, forming an efficient photoactive interface and significantly enhancing photocurrent signals [111]. These systems exemplify how smart hydrogels enable programmable, low-background, and enzyme- or target-responsive PEC detection. By coupling structural disassembly with photoactive release, such hydrogels offer powerful platforms for one-step, label-free, and reagentless PEC assays with amplified outputs and modular adaptability.

#### 2.2.3. Antifouling and Selectivity Control

In complex sample matrices, such as biological fluids, serum, or food samples, nonspecific adsorption of macromolecules (e.g., proteins) often results in electrode passivation and signal fluctuation, severely affecting the accuracy and reproducibility of PEC sensors. Hydrogels, owing to their hydrophilic and porous nature, act as selective physical barriers that prevent biofouling while allowing the unhindered diffusion of small target analytes and charge carriers [60,118,129]. The tunable porosity, charge density, and surface chemistry of hydrogels enable size- and charge-selective screening at the sensing interface, improving both selectivity and the signal-to-noise ratio of PEC outputs. For example, acrylamide-based hydrogels have been integrated with zwitterionic MOF layers to construct a multilevel screening interface. This structure effectively excluded interfering proteins while maintaining access for small molecules, like dopamine, ensuring reliable in vivo detection performance even in complex bioenvironments [118]. Moreover, hydrogel coatings can significantly enhance surface hydrophilicity and biocompatibility, further mitigating nonspecific adsorption. In wearable or implantable PEC systems, such hydrogel-modified surfaces also contribute to long-term operational stability. As a result, antifouling hydrogel strategies are increasingly favored in real-time and point-of-care PEC biosensing applications.

#### 2.2.4. In Situ Formation of Insulating Hydrogel for Signal-Off Regulation

Some hydrogel-based systems exploit the ability of target-triggered reactions to generate insulating films directly on the electrode surface. These hydrogels may form as a result of enzymatic or chemical crosslinking events initiated by the analyte itself or a related intermediate. Once formed, the hydrogel physically blocks electron transfer between the photoactive material and the electrolyte, thereby suppressing the photocurrent in a target-dependent manner [108,130,131]. For example, alginate hydrogels formed by Ca^2+^-induced gelation have been used to regulate drain current in organic PEC transistor platforms, achieving target-responsive signal-off detection of proteins such as HigG [130]. Similarly, a supramolecular MnO_2_-doped hydrogel was constructed to modulate interfacial light absorption and carrier diffusion, enabling efficient photocurrent suppression in Human epidermal growth factor receptor 2 (HER2) biosensing [131]. This in situ hydrogel-blocking strategy offers a robust and quantifiable readout mechanism, particularly valuable for binary-response or threshold-type PEC assays.

#### 2.2.5. Flexible Electrolyte and Polarization Medium

Beyond their role as structural matrices, hydrogels can also function as ionic conductors, replacing conventional liquid electrolytes in solid-state PEC configurations [69,109]. Their ionic conductivity supports efficient charge transport, while their gel consistency prevents leakage and enables conformal contact with curved or deformable surfaces. In some systems, hydrogels also contribute to photoresponse via light-induced dipole polarization of water molecules. For instance, an Fe^3+^-crosslinked PEDOT/alginate hydrogel was employed in a flexible PEC photodetector, where water dipole alignment under illumination generated transient photocurrents without traditional semiconductors [127]. Such polarization-driven photoresponses offer new avenues for bias-free and transparent PEC sensing platforms. These properties make hydrogel electrolytes especially attractive for developing wearable PEC devices and self-powered sensors.

#### 2.2.6. Visual Output and Multi-Functional Integration

Hydrogels can be readily combined with electrochromic compounds, photonic elements, or imaging interfaces to produce visible changes in color or brightness in response to PEC events [69,132]. This integration enables intuitive, label-free output signals suitable for point-of-care testing and portable field applications. The multifunctional nature of hydrogels allows them to simultaneously participate in light harvesting, signal conversion, and visual reporting. For instance, hydrogels with visual responsiveness have been employed in photoelectrochromic biosensors. In such systems, PEC reactions drive electrochromic transformations within the hydrogel matrix, generating ratiometric visual signals [132]. Additionally, colored gelatin-based hydrogels have been applied in PEC logic gates and retinomorphic synapses, mimicking cone cell responses to RGB stimuli [69]. These hydrogels not only function as chromogenic sensors but serve as programmable gates for visual information encoding and storage. Overall, the integration of hydrogels with optical signal reporters and microfabrication-compatible substrates expands the design possibilities of PEC systems, enabling real-time visualized sensing, multifunctional diagnostics, and intelligent array-based platforms.

## 3. Applications of Hydrogel-Based PEC Sensors

The versatility of hydrogel-based PEC sensors has enabled their application across a broad spectrum of analytes, ranging from simple ions and small organic molecules to complex biomacromolecules and living systems. Depending on the type of hydrogel used and the sensing strategy employed, these platforms have demonstrated excellent sensitivity, selectivity, and adaptability in diverse fields, such as environmental monitoring, food safety, medical diagnostics, and wearable bioelectronics. In the following sections, we classify and discuss recent representative applications of hydrogel-assisted PEC sensors according to the nature of the target analytes, highlighting key performance metrics, sensing mechanisms, and real-sample demonstrations.

### 3.1. Detection of Ions

**Figure 2:**
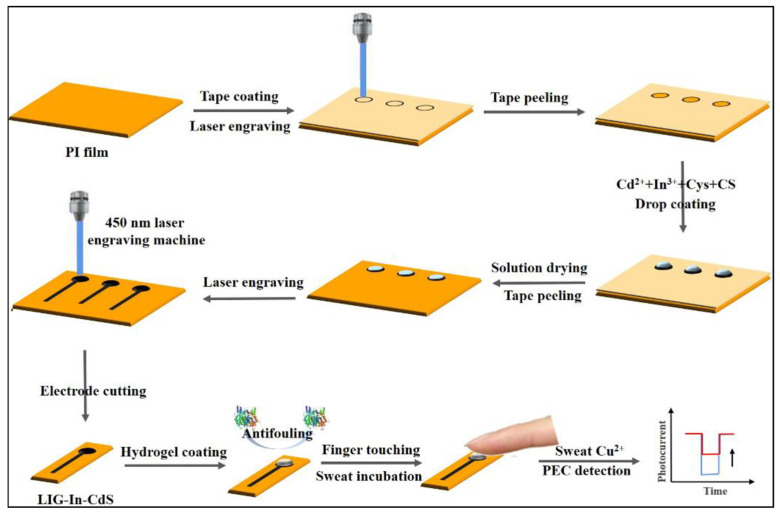
Schematic illustration of the PEC sensor based on a polyvinyl alcohol (PVA) antifouling hydrogel layer for the detection of Cu^2+^ ions. Reproduced with permission from reference [60].

To date, hydrogel-assisted PEC sensors have shown promise in detecting metal ions, with particular advances in sensing copper (Cu^2+^) and ruthenium (Ru^3+^). Wang et al. reported a wearable PEC sensor based on laser-induced graphene (LIG) integrated with In-doped CdS (LIG-In-CdS) for noninvasive detection of Cu^2+^ in natural human sweat (Figure 2) [60]. The photoelectrode is fabricated using a cost-effective 450 nm laser to engrave a chitosan-based precursor film containing cadmium and indium salts on a polyimide substrate, forming a porous, conductive, and photoactive structure. A porous polyvinyl alcohol (PVA) hydrogel patch is employed as a sweat collector, providing excellent antifouling performance while maintaining compatibility with real-time Cu^2+^ detection. Upon contact with sweat, Cu^2+^ binds to CdS to form Cu_x_S, which increases carrier recombination and decreases the photocurrent, establishing a signal-off PEC detection mode. The sensor achieves a linear response range of 1.28 ng∙mL^−1^ to 5.12 μg∙mL^−1^, and its performance was validated in both natural and exercise-induced sweat, showing excellent agreement and recovery values between 98.1% and 102.9%. The simple “touch–incubate–detect” workflow, high sensitivity, and excellent selectivity make this PEC system promising for wearable biosensing platforms.

Building on the theme of hydrogel-templated interfaces, subsequent research has leveraged hydrogel-mediated synthesis strategies to fabricate novel functional materials. In a separate study, Liu and Wang et al. developed a confined synthesis strategy for free-standing two-dimensional covalent organic framework (2D COF) thin films using a superspreading water layer formed on immersed polyacrylamide (PAAm) hydrogels [133]. A Schiff-base condensation reaction between 4,4′,4″-(1,3,5-triazine-2,4,6-triyl)trianiline (TTA) loaded in the hydrogel and 2,5-dihydroxyterephthalaldehyde (DHTA) dissolved in the oil phase led to the formation of crystalline COFTTA DHTA films with tunable thickness (4–150 nm) and uniform surface morphology. The COF films displayed an ordered hexagonal pore structure (ca. 3.4 nm), excellent crystallinity, and remarkable mechanical strength (Young’s modulus ~25.9 GPa). When transferred to ITO substrates, the films demonstrated strong PEC activity. The imine linkages in the COF frameworks served as coordination sites for Ru^3+^, enabling complex formation that significantly enhanced the photocurrent. The PEC response exhibited a linear relationship with Ru^3+^ concentration in the range of 0.3–30 μM, with excellent selectivity over competing metal ions. In addition, the COF thin films functioned as selective nanofiltration membranes, effectively rejecting nanoparticles (e.g., 4 nm AuNPs) while allowing small dye molecules to pass. This approach not only offers a scalable route to high-quality COF films but demonstrates potential in dual-function PEC sensing and size-selective molecular separation.

### 3.2. Detection of Small Molecules

Following the ion detection section, which highlighted the hydrogel’s advantages in antifouling and ion-selective transport, this section focuses on the detection of small organic molecules. These targets, such as neurotransmitters, metabolic intermediates, drugs, and pesticides, play critical roles in physiological and pathological processes and often exist in complex biological or environmental matrices, posing challenges for PEC analysis.

#### 3.2.1. Biomolecules

Biological small molecules play crucial roles in maintaining physiological homeostasis and mediating biochemical signaling. Representative examples include neurotransmitters, reactive oxygen species, metabolic intermediates, and nucleic acid components. Abnormal fluctuations in the concentration of these molecules are often associated with pathological conditions, such as neurodegenerative disorders, cancer, diabetes, and oxidative stress-related diseases. Therefore, the accurate, real-time, and non-invasive detection of these biomarkers is of great significance for early diagnosis, therapeutic monitoring, and personalized medicine [134,135,136,137,138,139,140]. Hydrogel-based PEC sensors offer distinct advantages in detecting such analytes due to their high biocompatibility, tunable porosity, and ability to support selective recognition and signal amplification. By serving as antifouling layers, molecular immobilization matrices, or responsive transduction media, hydrogels have been effectively integrated into PEC platforms for the sensitive and selective analysis of dopamine, H_2_O_2_, glucose, sarcosine, lactate, guanine, and related targets. In the following section, representative sensor designs and analytical strategies for these molecules are summarized, highlighting the role of hydrogel structures in enhancing performance and enabling real-sample applicability.

**Figure 3:**
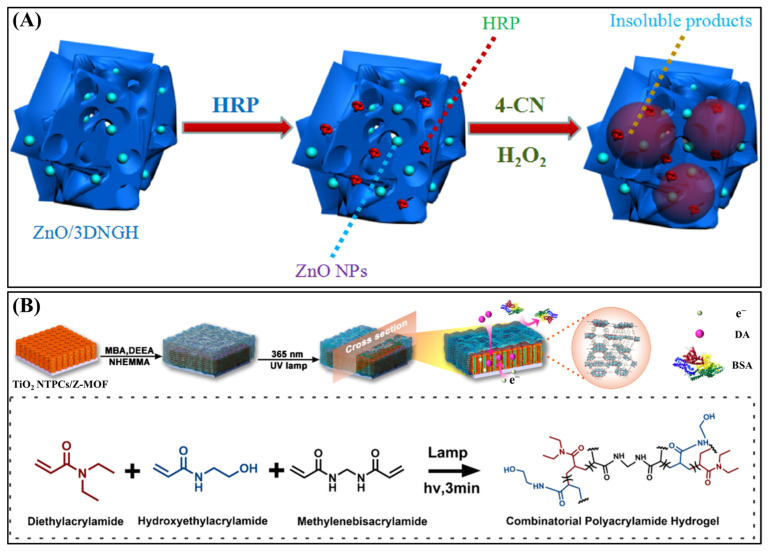
(**A**) Schematic illustration of the PEC sensing mechanism for H_2_O_2_ based on ZnO nanoparticles embedded in a nitrogen-doped three-dimensional graphene hydrogel (3DNGH) coupled with horseradish peroxidase (HRP). Reproduced with permission from reference [141]. (**B**) Fabrication and signal transduction diagram of the TiO_2_ NTPCs/Z-MOF/hydrogel PEC biosensor for dopamine detection under antifouling conditions. Reproduced with permission from reference [118].

Among recent developments, the use of enzyme-loaded hydrogel scaffolds has enabled dual-analyte detection in biomedical and food systems. Wang et al. constructed a high-performance PEC sensor by integrating ZnO nanoparticles with a nitrogen-doped three-dimensional graphene hydrogel (3DNGH), enabling efficient enzyme immobilization, light harvesting, and electron transport (Figure 3A) [141]. In this platform, H_2_O_2_ was detected via a signal-off mechanism in which horseradish peroxidase (HRP) catalyzed the oxidation of 4-chloro-1-naphthol into an insoluble insulating product, suppressing photocurrent generation. For glucose detection, a bienzyme cascade (GOx-HRP) was employed, generating H_2_O_2_ as an intermediate to trigger a similar current drop. The sensor demonstrated wide dynamic ranges (1 μM to 5 mM for H_2_O_2_ and 2 μM to 8 mM for glucose), low detection limits (0.33 and 0.66 μM), and was successfully validated in fruit juice samples, confirming its applicability for both biomedical and food analysis contexts.

To address the challenges in in vivo electrochemical monitoring, soft hydrogel-based microelectrodes have also been employed. Zhang et al. developed a soft, biocompatible hydrogel-based PEC microelectrode incorporating in situ-grown AgInS_2_ quantum dots for dopamine (DA) monitoring under visible light [107]. The conductive hydrogel matrix, formed from acrylamide and polyrotaxane-poly(ethylene glycol) methacrylate (PEGMA), was doped with PEDOT/PSS to match the mechanical softness and conductivity of brain tissue. DA oxidation triggered a positive feedback loop involving polydopamine formation and ROS-mediated enhancement of photocurrent quenching. This enabled ultrasensitive real-time analysis with a detection limit of 64 nM and high selectivity. The implantable sensor successfully tracked DA fluctuations in the mouse striatum without inducing inflammation, validating its potential for in vivo neurochemical monitoring. In a complementary study, the same research group reported a dual-layer antifouling PEC sensor that combined zwitterionic MOFs (Z-MOFs) with a polyacrylamide hydrogel to selectively detect DA in complex media (Figure 3B) [118]. Built upon a TiO_2_ nanotube photonic crystal substrate, the sensor utilized sulfonated MIL-125 as a hydrophilic molecular sieve and overlaid it with a hydrogel to enable free diffusion of small molecules. DA coordination to Ti^4+^ sites enhanced the photocurrent, yielding a linear range from 10 to 500 nM and a detection limit of 6.0 nM. The antifouling architecture maintained performance even in protein-rich environments and demonstrated excellent biocompatibility, underscoring its potential for long-term in vivo sensing.

Building on hydrogel programmability, a DNA hydrogel-based architecture has been explored to enhance glucose sensing via enzyme cascade amplification. Tang et al. introduced a signal-off PEC platform for glucose detection by integrating an In_2_O_3_–CdS nanocomposite with a DNA hydrogel encapsulating glucose oxidase (GOx) and HRP [113]. Upon glucose oxidation, enzymatically produced H_2_O_2_ initiated a bromide-assisted bioetching reaction of CdS, decreasing the photocurrent through suppressed electron transport. The DNA hydrogel provided structural integrity, enhanced enzyme activity, and allowed programmable architecture. The biosensor exhibited a detection range from 10 nM to 10 μM and a detection limit of 9.62 nM. It also showed strong selectivity against sugars and proteins, offering robust performance for bioanalytical diagnostics. To further expand the sensing range to multiple metabolites, light-addressable hydrogel arrays have been demonstrated. Wang, Bu, and Zhang et al. proposed a multiplexed PEC biosensing array based on light-addressable electrochemistry (LAE), using a silicon photoelectrode functionalized with a Fc-containing PEG–gelatin hydrogel [128]. This hydrogel immobilized both redox mediators and enzymes without leakage, enabling the stable detection of glucose, sarcosine, and lactate. Upon illumination, enzymatic consumption of Fc^+^ modulated the reduction photocurrent in an analyte-dependent manner. Each sensing region was activated by targeted laser scanning. The platform delivered detection limits of 179 μM (glucose), 16 μM (sarcosine), and 780 μM (lactate), and showed excellent recovery in human sweat samples. This versatile system demonstrates potential for wearable diagnostics and real-time metabolic monitoring.

**Figure 4:**
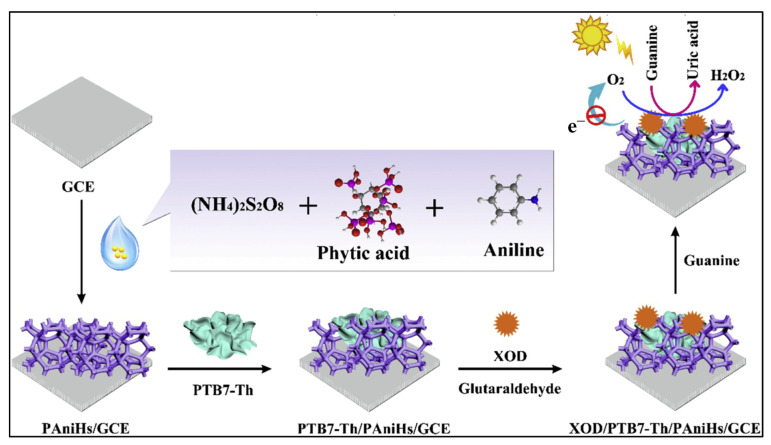
Enzymatic photoelectrochemical sensor based on a 3D PTB7–Th/polyaniline hydrogel (PAniHs) composite for guanine detection. Reproduced with permission from reference [117].

In addition to oxidizable targets, nucleobases such as guanine have been detected using redox-active hydrogel supports. Luo and Li et al. developed a photocathodic PEC biosensor based on a donor–acceptor conjugated polymer (PTB7-Th) embedded in a 3D polyaniline hydrogel (PAniHs), tailored for guanine detection (Figure 4) [117]. The hydrogel provided a porous and conductive scaffold for enzyme immobilization and enhanced photoelectronic interactions. Xanthine oxidase (XOD) catalyzed the conversion of guanine to uric acid, consuming O_2_—a key electron acceptor—thereby reducing the cathodic photocurrent. The system achieved a broad detection range (0.1–80 μM), a detection limit of 20 nM, and successful application in real sample analysis with recovery rates between 91.0% and 108.8%. This sensitizer-free, environmentally friendly sensor presents a robust platform for nucleobase analysis and oxidative stress evaluation. Furthermore, hydrogel-immobilized redox enzymes have also been used to visualize hydrogen generation at high spatial resolution. Schuhmann et al. introduced a microscale PEC biosensor for hydrogen detection based on [NiFe]-hydrogenase (H_2_ase) immobilized within a viologen-functionalized redox hydrogel [142]. Integrated onto carbon microelectrodes and coupled with scanning photoelectrochemical microscopy (SPECM), the system enabled spatially resolved measurement of H_2_ generated from photocatalytic substrates. Hydrogen oxidation by the enzyme transferred electrons via viologen moieties to the electrode, producing a photocurrent detectable at 0 mV vs. Ag/AgCl. The biosensor showed a detection limit of 0.81 μM and excellent signal contrast compared to platinum-based systems. Its high spatial resolution makes it a valuable tool for visualizing local photo(bio)catalytic activity and evaluating hydrogen-evolving materials.

#### 3.2.2. Drug and Pesticide Molecules

Drug residues and pesticide contaminants are of major concern in biomedical and environmental safety due to their bioaccumulation, persistence, and potential toxicity. Small-molecule analytes, such as antibiotics and pesticides, are frequently monitored in food, water, and biological tissues [143,144,145,146,147,148,149]. PEC sensors enable label-free, portable, and real-time detection of such pollutants with improved antifouling capability and biocompatibility. The following studies exemplify the design and application of such hydrogel-integrated PEC platforms for the detection of pharmaceutical and agrochemical targets.

**Figure 5:**
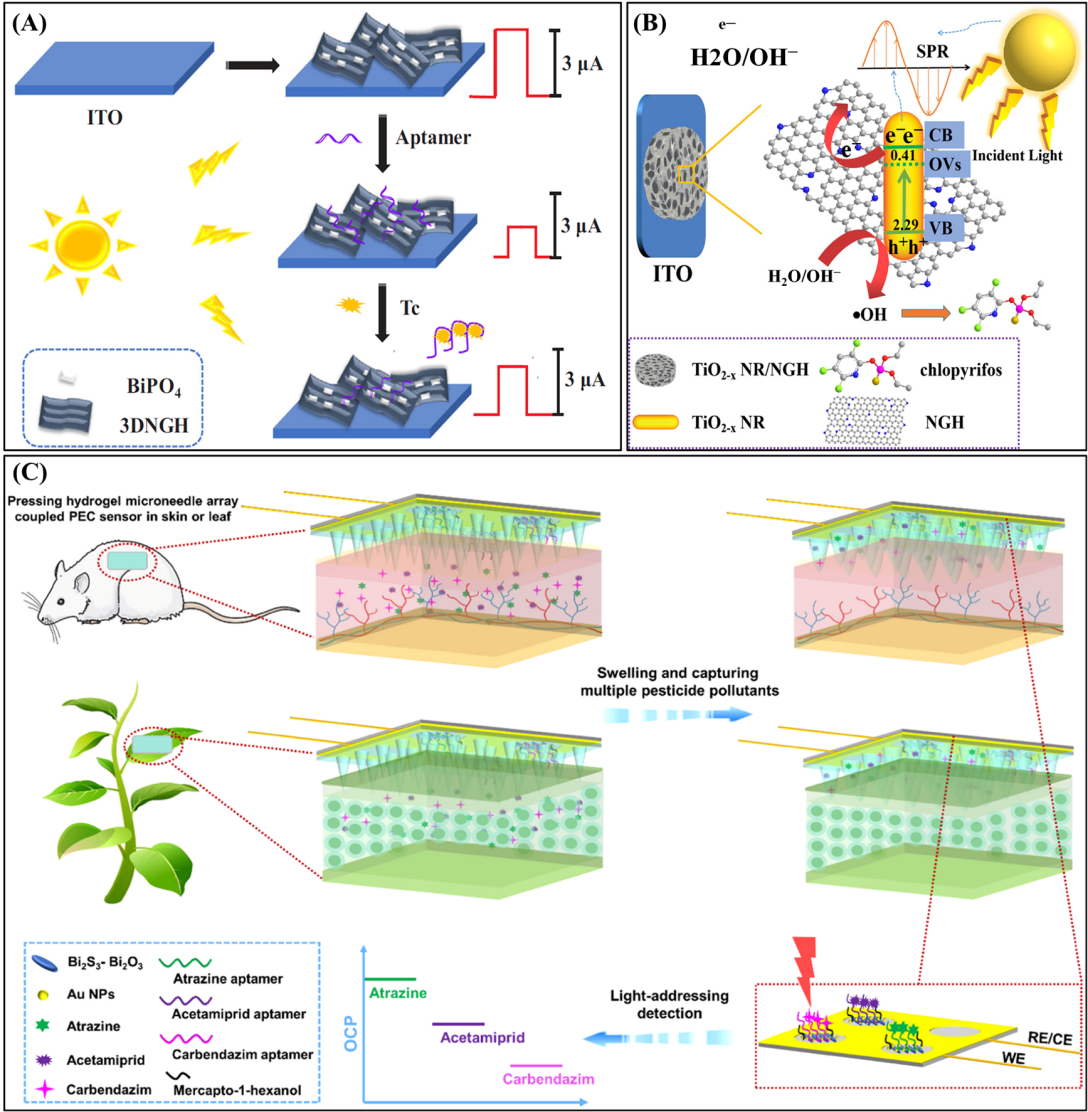
(**A**) Schematic illustration of a PEC aptasensor based on a BiPO_4_/three-dimensional nitrogen-doped graphene hydrogel (3DNGH) composite for tetracycline (Tc) detection. Reproduced with permission from reference [124]. (**B**) PEC sensor constructed using self-doped Ti^3+^ nanorods (TiO_2−x_) integrated with a three-dimensional nitrogen-doped graphene hydrogel (NGH) for the detection of chlorpyrifos. Reproduced with permission from reference [121]. (**C**) Bi_2_S_3_–Bi_2_O_3_ photoelectrode with a swellable hydrogel microneedle array based PEC sensor for multiple detection of atrazine (ATZ), acetamiprid (ACP), and carbendazim (CBZ). Reproduced with permission from reference [129].

Liu and Wang et al. developed a visible-light-driven PEC aptasensor for the ultrasensitive detection of tetracycline (Tc), utilizing a one-pot synthesized BiPO_4_/three-dimensional nitrogen-doped graphene hydrogel (3DNGH) composite (Figure 5A) [124]. The 3DNGH framework significantly improves the charge transport and visible-light utilization of BiPO_4_ nanorods, narrowing the bandgap from 3.85 eV to 2.10 eV and enhancing photocurrent generation under visible light. A label-free PEC aptasensor was constructed by immobilizing a Tc-specific aptamer onto the BiPO_4_/3DNGH-modified ITO electrode. The aptamer hinders electron transfer via π–π stacking, resulting in a low background photocurrent. Upon Tc binding, the aptamer is released, reducing interfacial resistance and increasing the photocurrent, enabling sensitive detection. The sensor exhibits a wide linear detection range (0.1 nM–1 μM) with a low limit of detection of 0.033 nM. When applied to milk samples, it achieved recovery rates of 98.0–105.3%, demonstrating high accuracy and applicability in food safety monitoring. The Wang group later constructed a label-free PEC aptasensor based on a MoS_2_/nitrogen-doped graphene hydrogel (NGH) p–n heterojunction for chloramphenicol (CAP) detection [125]. The hybrid material featured enhanced electron–hole separation and visible-light photoactivity. CAP-specific aptamers were anchored via π–π interactions. Upon binding, CAP underwent photooxidation, increasing the photocurrent. The sensor showed a linear range of 32.3 ng∙L^−1^ to 96.9 μg∙L^−1^ and a detection limit of 3.23 ng/L. It maintained specificity in honey matrices with recoveries ranging from 98.0% to 106.6%, confirming its robustness for real-sample applications.

To further leverage plasmonic enhancement effects, defect-engineered TiO_2_ and NGH composites have been employed for pesticide sensing. Du et al. reported a PEC sensor based on a non-noble metal plasmonic strategy for the sensitive detection of chlorpyrifos (Figure 5B) [121], a widely used organophosphate pesticide. The sensor integrated self-doped Ti^3+^ nanorods (TiO_2−x_) with a three-dimensional nitrogen-doped graphene hydrogel (NGH) to construct a synergistic 1D–3D heterostructure that improved charge transport, light absorption, and carrier separation. The PEC mechanism was governed by localized surface plasmon resonance (LSPR) effects originating from Ti^3+^ centers and oxygen vacancies within TiO_2−x_, facilitating efficient visible-light harvesting. Upon illumination, photoexcited holes in the TiO_2−x_ valence band generated hydroxyl radicals (∙OH), which oxidized chlorpyrifos and enhanced the photocurrent response. The system exhibited a wide linear range from 0.05 ng∙mL^−1^ to 0.5 µg∙mL^−1^ and achieved an ultralow detection limit of 0.017 ng∙mL^−1^. The sensor exhibited excellent selectivity and stability in wastewater analysis.

In addition to surface sensors, implantable hydrogel fiber platforms have been developed for the long-term in vivo monitoring of toxicants. Wang et al. introduced an implantable PEC hydrogel fiber sensor for long-term in vivo detection of pentachlorophenol (PCP) [150]. The fiber comprises a polyethylene glycol diacrylate (PEGDA) hydrogel matrix embedded with PCN-224(Zn)@TiO_2_ and Au@Ag nanowires, further coated with calcium alginate for antifouling. The axial coordination between PCP and Zn centers suppressed photoinduced electron transfer, reducing the photocurrent. This dual-mode PEC/fluorescence system exhibited a wide dynamic range (0.05–1000 ng∙mL^−1^) and an ultralow limit of detection (LOD) of 2.9 fg∙mL^−1^. Implanted in free-swimming fish brains, the sensor tracked PCP for 70 days without inducing inflammation, underscoring its value in ecological monitoring. To further minimize invasiveness while enabling multi-target analysis, microneedle-based PEC devices have emerged as a powerful strategy. In a further study, Wang et al. advanced a minimally invasive PEC sensor integrating a Bi_2_S_3_–Bi_2_O_3_ photoelectrode with a swellable hydrogel microneedle array (Figure 5C) [129]. This platform enabled the multiplexed detection of atrazine (ATZ), acetamiprid (ACP), and carbendazim (CBZ) in skin and plant tissues. Specific aptamers functionalized each sensing zone on a single ITO electrode. Target binding reduced open-circuit potential, establishing a signal-off response. The system achieved detection limits as low as 0.029 fg∙mL^−1^ (ATZ), 5.5 fg∙mL^−1^ (ACP), and 21 fg∙mL^−1^ (CBZ). It demonstrated rapid swelling, biocompatibility, and minimal invasiveness, supporting longitudinal monitoring in vivo.

#### 3.2.3. Mycotoxins and Pollutants

Mycotoxins, environmental pollutants, and related hazardous chemicals pose serious risks to human health and ecological systems, even at ultratrace levels [151,152,153,154,155,156]. Hydrogel-integrated PEC sensors provide an ideal platform due to their high sensitivity, structural adaptability, and compatibility with flexible, portable formats. The following examples illustrate recent progress in hydrogel-based PEC platforms for the detection of these hazardous substances across environmental and food matrices.

**Figure 6:**
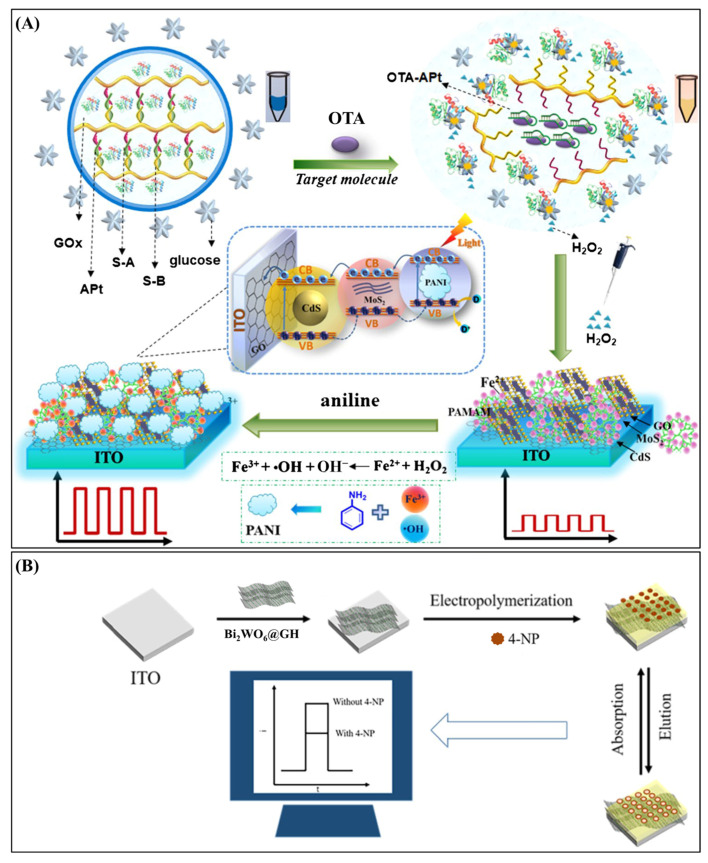
(**A**) Schematic illustration of a PEC sensor for ochratoxin A (OTA) detection based on a responsive DNA hydrogel and in situ polymerization of polyaniline for signal amplification. Reproduced with permission from reference [112]. (**B**) PEC sensor constructed using three-dimensional (3D) structured Bi_2_WO_6_@graphene hydrogel (GH) composites for the detection of 4-nitrophenol. Reproduced with permission from reference [123].

For rapid, field-deployable testing, visual PEC biosensors integrated with electrochromic hydrogels have been designed. Wang et al. developed a portable solar-powered ratiometric photo-electrochromic biosensor for the visual detection of ochratoxin A (OTA) [132]. The sensor integrated a Co,N-doped TiO_2_/three-dimensional graphene hydrogel (Co,N-TiO_2_/3DGH) as the photoactive material and Prussian blue (PB) as the electrochromic color indicator, both deposited onto separate regions of a single indium tin oxide (ITO) electrode. Upon visible light exposure, photogenerated electrons from the Co,N-TiO_2_/3DGH layer reduced PB (from blue to white). The binding of OTA to the aptamer-modified sensing region increased steric hindrance, which impeded electron transfer and slowed PB decoloration. To ensure accurate quantification under variable sunlight conditions, a reference module was incorporated, and detection was based on the chromaticity ratio between the sensing and reference regions. The biosensor exhibited a linear detection range of 1–500 ng/mL and a detection limit of 0.29 ng/mL. When tested in corn juice samples, it achieved high recovery rates (98.0–101.2%) and excellent reproducibility (relative standard deviation, RSD: 2.33–3.36%), with results closely aligned with HPLC validation. Its instrument-free operation, affordability, and rapid response made it highly suitable for field-based food safety screening and environmental monitoring. By contrast, stimuli-responsive DNA hydrogels have enabled signal amplification in flexible PEC sensors for food contaminants. Tang et al. developed a flexible, label-free PEC biosensor for the detection of OTA, which integrated a DNA hydrogel with an MoS_2_–CdS heterostructure functionalized by poly(amidoamine) (PAMAM)-Fe^2+^ (Figure 6A) [112]. The DNA hydrogel, formed by aptamer-crosslinked strands, encapsulated glucose oxidase (GOx). Target binding triggered hydrogel collapse, resulting in the release of GOx, which subsequently catalyzed the oxidation of glucose to produce H_2_O_2_. This triggered a Fenton-like reaction that oxidized aniline into polyaniline (PANI), which deposited onto the photoelectrode and boosted the photocurrent. The system achieved an ultralow detection limit of 0.05 pg/mL with excellent recovery (98.4–102.9%) in red wine. This smart hydrogel amplification mechanism broadens the scope for signal enhancement strategies.

To realize self-powered PEC systems based on Schottky interfaces for water toxin sensing, Jiang and Chen reported a self-powered PEC aptasensor based on a nitrogen-doped graphene hydrogel (NGH) and Fe_2_O_3_ nanocomposite for the ultrasensitive detection of microcystin-LR (MC-LR) under visible light [120]. The NGH/Fe_2_O_3_ heterojunction, synthesized via a one-pot hydrothermal method, formed a Schottky interface that significantly enhanced charge separation and increased carrier density, resulting in superior photocurrent output. An aptamer specific to MC-LR was immobilized on the NGH/Fe_2_O_3_-modified electrode to enable selective molecular recognition. Upon target binding, the aptamer underwent a conformational change that facilitated electron transfer and amplified the photocurrent, achieving a “signal-on” detection mode without requiring external bias. The sensor exhibited a wide linear detection range from 1 pM to 5 nM and achieved an exceptionally low detection limit of 0.23 pM—well below the World Health Organization’s guideline for drinking water. It also demonstrated high selectivity over structurally similar toxins and pesticides, along with excellent operational stability. When applied to real water samples, the aptasensor delivered recovery rates ranging from 96% to 104%. This study presented a promising strategy for environmental toxin detection, leveraging Schottky interface design and 3D conductive hydrogel frameworks to enable efficient, low-cost PEC biosensing under ambient conditions.

Finally, for airborne pollutant monitoring, molecularly imprinted hydrogels combined with 3D structured semiconductors enable highly selective PEC analysis. Yao and Xu et al. developed a high-performance photoelectrochemical (PEC) sensor for the trace detection of 4-nitrophenol (4NP) in PM2.5, based on dual three-dimensional (3D) structured Bi_2_WO_6_@graphene hydrogel (GH) composites modified with molecularly imprinted polypyrrole (MIPs) (Figure 6B) [123]. A hydrothermal method was employed to uniformly anchor flower-like Bi_2_WO_6_ microspheres onto the conductive 3D GH framework, yielding a hybrid material with enhanced light-harvesting capability, charge transport efficiency, and high surface area. Molecularly imprinted PPy films were electropolymerized onto the Bi_2_WO_6_@GH-coated ITO electrode, introducing selective recognition sites tailored to the shape and functional groups of 4NP. Upon visible-light irradiation, photogenerated electrons from Bi_2_WO_6_ were transferred through the GH network to generate a measurable photocurrent. When 4NP molecules reoccupied the imprinted cavities, electron transport was hindered, and charge recombination was promoted, resulting in a signal-off PEC response. Under optimized conditions, the sensor exhibited a broad linear detection range from 5.0 × 10^−12^ to 1.0 × 10^−7^ M and an ultralow detection limit of 5.78 × 10^−13^ M. It demonstrated excellent selectivity against structurally similar interferents, high reproducibility, and long-term operational stability. The system was successfully applied to the analysis of 4NP in real PM2.5 samples, underscoring its potential for the environmental monitoring of airborne toxicants.

### 3.3. Detection of Biomacromolecules

Biomacromolecules, including microRNAs, proteins, and enzymes, are vital players in regulating cellular activities and maintaining physiological balance. Their aberrant expression or activity is often closely linked to the onset and progression of various diseases, making them essential biomarkers for clinical diagnostics and therapeutic monitoring. Hydrogel-based PEC sensors offer a powerful and versatile platform for detecting such biomacromolecules, owing to their excellent biocompatibility, structural tunability, and ability to support specific molecular recognition and signal amplification. In this section, we focus on recent advances in hydrogel-integrated PEC strategies for the sensitive and selective detection of three major classes of biomacromolecules: microRNAs, proteins, and enzymes.

#### 3.3.1. microRNAs

MicroRNAs (miRNAs) are a class of endogenous, non-coding single-stranded RNA molecules, typically 18–24 nucleotides in length, that regulate gene expression post-transcriptionally by binding to the 3′ untranslated regions of target mRNAs. They are intricately involved in controlling diverse biological processes, such as cell proliferation, differentiation, apoptosis, and immune responses. Dysregulation of miRNA expression is frequently associated with a variety of pathological conditions, including cancers, cardiovascular diseases, and neurodegenerative disorders. Given their critical regulatory roles and disease relevance, miRNAs have emerged as valuable diagnostic and prognostic biomarkers [157,158,159,160,161,162]. However, their small size, low abundance, and sequence similarity pose significant challenges for accurate and sensitive detection, thereby necessitating the development of robust sensing technologies. Hydrogel-based PEC biosensors provide a powerful solution for miRNA analysis, combining the merits of hydrogel materials—such as programmability, biocompatibility, and responsiveness—with the high sensitivity and low background of PEC readouts. Recent studies have successfully exploited responsive DNA hydrogels, CRISPR-associated systems, and nanostructured photoactive materials to construct innovative PEC platforms for miRNA detection.

Wang et al. developed an innovative PEC biosensor capable of simultaneously detecting two microRNA targets—miRNA-141 and miRNA-21—by employing voltage-resolved signal differentiation [163]. The sensing platform integrated two CdTe-based nanocomposites: anodic CdTe-loaded carbon nitride nanosheets (CdTe–C_3_N_4_) and cathodic CdTe-loaded three-dimensional graphene hydrogel (CdTe–3DGH). These photoactive materials exhibited distinct potential-resolved photocurrent responses at −0.109 V and +0.27 V, respectively, allowing dual-analyte detection on a single ITO electrode without signal interference. The detection mechanism relied on competitive hybridization. In the absence of target miRNAs, complementary DNA-modified gold nanoparticles (cDNA–AuNPs) hybridized with immobilized probes, enhancing the photocurrent via plasmonic resonance. Upon target binding, the miRNAs displaced the cDNA–AuNPs, decreasing the signal intensity. The photocurrent changes were inversely proportional to the concentration of the corresponding miRNAs. The sensor achieved ultralow detection limits of 0.63 fM for miRNA-141 and 0.29 fM for miRNA-21, with wide linear dynamic ranges up to 10^4^ and 10^5^ fM, respectively. It also demonstrated excellent selectivity against mismatched sequences, high reproducibility, and reliable performance in spiked serum samples, indicating its strong potential for clinical diagnostics, cancer biomarker analysis, and miRNA-related disease monitoring.

To address the challenge of integrating recognition and amplification elements within a single matrix, researchers have also explored homogeneous PEC systems employing target-responsive hydrogels. Guo and Lin et al. developed a homogeneous PEC biosensor for the sensitive detection of microRNA-155 using a target-responsive DNA hydrogel as both the recognition and signal amplification unit [114]. The hydrogel was formed through crosslinking hyaluronic acid with polyethylenimine and TiO_2_-labeled DNA duplexes. Upon hybridization with miRNA-155, a cascade reaction involving Exo III and Nb.BbvCI endonuclease triggered specific hydrogel disassembly and the release of embedded TiO_2_ nanoparticles. These nanoparticles, when free in solution, acted as photoactive elements and produced an enhanced photocurrent under visible-light illumination. The assay operated without electrode modification, enabling simple and reproducible measurements. It achieved a detection limit as low as 0.41 fM and exhibited excellent selectivity among homologous miRNA sequences. Furthermore, the platform demonstrated effective performance in complex biological environments, including lysates from breast cancer cell lines, validating its utility for real biological sample analysis. This work highlighted the potential of integrating hydrogel responsiveness with enzyme-assisted signal release for highly sensitive, homogenous PEC biosensing.

**Figure 7:**
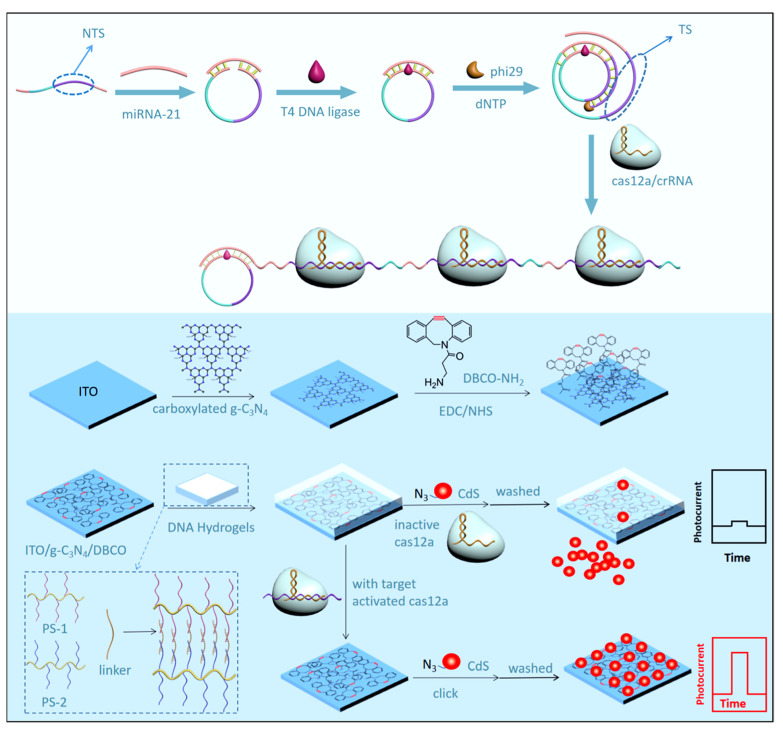
Schematic illustration of the PEC biosensor for miRNA-21 detection based on a responsive DNA hydrogel and target-triggered activation of the CRISPR/Cas12a system. Reproduced with permission from reference [111].

In addition to enzymatic hydrogel disassembly mechanisms, other designs have incorporated gene-editing machinery, such as CRISPR/Cas systems, to achieve programmable and ultrahigh sensitivity. Zhang et al. proposed a label-free PEC biosensor for miRNA-21 detection by integrating a CRISPR/Cas12a system with a DNA hydrogel-gated photoelectrode interface (Figure 7) [111]. The detection strategy utilized rolling circle amplification (RCA) to generate long ssDNA sequences upon miRNA-21 recognition. These products subsequently activated the collateral cleavage function of Cas12a, which degraded a DNA hydrogel that originally blocked the interaction between azide-functionalized CdS quantum dots (CdS-N_3_) and g-C_3_N_4_/ITO electrodes bearing dibenzocyclooctyne (DBCO) groups. Once the hydrogel barrier was removed, a bioorthogonal click reaction occurred, forming a CdS/g-C_3_N_4_ heterojunction that significantly enhanced the photocurrent. This unique signal-on design allowed for ultrahigh sensitivity, achieving a detection limit of 3.2 aM. The sensor exhibited high selectivity, distinguishing target miRNA from single-base mismatches. It also performed reliably in human serum, underscoring its practical application potential. The study demonstrated a modular and programmable PEC detection strategy that combines the specificity of CRISPR technology with the functional versatility of DNA hydrogels.

#### 3.3.2. Proteins

Proteins are complex macromolecules composed of one or more polypeptide chains folded into specific three-dimensional structures. They serve a vast array of biological functions, including catalysis (enzymes), signaling (hormones), structural support (collagen), immune defense (antibodies), and transport (hemoglobin). Due to their fundamental role in cellular physiology and disease pathology, the sensitive and specific detection of proteins—particularly disease biomarkers, such as carcinoembryonic antigen (CEA), prostate-specific antigen (PSA), and HER2—is essential for early diagnosis, prognosis, and monitoring of therapeutic efficacy in conditions like cancer, cardiovascular diseases, and neurodegeneration [164,165,166,167,168]. Hydrogel-based PEC sensors have emerged as promising tools for protein detection. The hydrated, porous, and tunable structure of hydrogels facilitates bioreceptor immobilization, enhances antifouling properties, and enables responsive interface engineering. Below, six recent studies are summarized to illustrate diverse strategies integrating hydrogels into PEC platforms for protein sensing.

**Figure 8:**
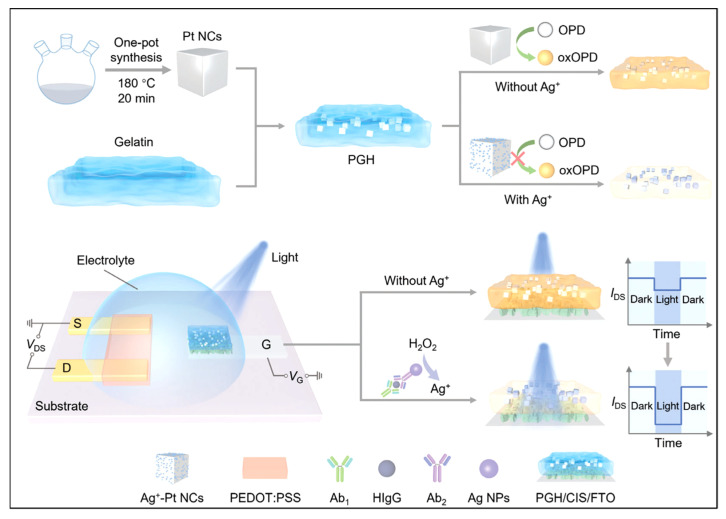
Schematic illustration of an organic photoelectrochemical transistor (OPECT) biosensor gated by a platinum nanocube-embedded gelatin hydrogel. Reproduced with permission from reference [108].

Zeng, Zhao, and Lin et al. developed a hydrogel/graphene oxide hybrid (HGH)-gated organic photoelectrochemical transistor (OPECT) for the ultrasensitive detection of human immunoglobulin G (HIgG) under zero gate bias [130]. The sensor employed a CdS quantum dot-coated ITO gate electrode, while the channel consisted of a PEDOT/PSS conducting polymer. Upon completion of a sandwich immunoassay, Ca^2+^ ions released from the immune complex triggered gelation of a sodium alginate/graphene oxide (SA/GO) mixture, forming an opaque hydrogel layer at the gate interface. This hydrogel significantly impeded both light penetration and ionic conductivity, thereby modulating the drain current (I_DS_) through photogating effects. The OPECT device demonstrated a wide detection range from 100 fg∙mL^−1^ to 100 ng∙mL^−1^ with an ultralow limit of detection of 50 fg∙mL^−1^. The system exhibited strong specificity against common biological interferents and maintained excellent recovery rates in spiked serum samples. This design showcased a scalable, low-power PEC sensing platform capable of high sensitivity without external gate voltages. In a subsequent study, the same group introduced a novel color-gated OPECT biosensor, integrating a platinum nanocube-embedded gelatin hydrogel (PGH) as the optical gate (Figure 8) [108]. In this system, Pt nanocubes catalyzed the oxidation of o-phenylenediamine (OPD), yielding a colored product whose intensity was modulated by Ag^+^ ions. During a sandwich immunoassay for HIgG, silver nanoparticles labeled on secondary antibodies released Ag^+^, which inhibited Pt catalytic activity and thus reduced OPD oxidation. The resulting lighter hydrogel color allowed greater light transmittance to the CIS/FTO photoelectrode, enhancing the photogating efficiency and modulating I_DS_ in the PEDOT/PSS channel. This platform achieved a detection range from 10 fg∙mL^−1^ to 10 ng∙mL^−1^ with a detection limit of 10 fg∙mL^−1^, along with high selectivity and reliable performance in human serum. Importantly, this work established a new class of visually regulated OPECTs that synergize optical and electronic signals, offering a promising approach for self-powered biosensing and next-generation optoelectronic diagnostics.

**Figure 9:**
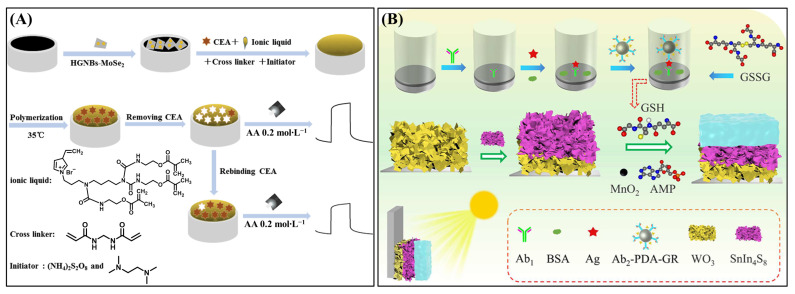
(**A**) Schematic illustration of the molecularly imprinted polymer (MIP) hydrogel sensor for carcinoembryonic antigen (CEA) detection. Reproduced with permission from reference [115]. (**B**) Schematic illustration of the PEC immunosensor for HER2 detection. Target-triggered formation of a MnO_2_-doped supramolecular hydrogel on the WO_3_/SnIn_4_S_8_ heterojunction inhibits photocurrent by introducing steric hindrance and competing for incident light. Reproduced with permission from reference [131].

While transistor-based systems focus on optical-electronic integration, molecular imprinting techniques have also been used to engineer artificial recognition interfaces. Li et al. reported a label-free PEC biosensor for the selective detection of CEA using a molecularly imprinted polymer (MIP) hydrogel (Figure 9A) [115]. The MIP was fabricated from a polymerized ionic liquid monomer (BCCPEimBr) on a photoactive interface composed of hollow gold nanoballs (HGNBs) and MoSe_2_ nanosheets. The hydrogel acted as both a biocompatible scaffold and a synthetic recognition matrix, preserving the native conformation of CEA during imprinting. Upon target binding, CEA molecules occupied the imprinted cavities, hindering charge transport and resulting in a photocurrent decrease. The sensor demonstrated a linear detection range from 0.05 to 5.0 ng∙mL^−1^ and a detection limit of 11.2 pg∙mL^−1^. It exhibited strong selectivity, good reproducibility, and excellent agreement with chemiluminescent immunoassay (CLIA) results in human serum, confirming its feasibility for clinical diagnostics without the use of antibodies. In contrast to imprinting-based strategies, hydrogel-coated photoactive substrates have also enabled regenerable immunosensing with antifouling capacity. Wang and Kang et al. designed a regenerable, antifouling PEC immunosensor for detecting cardiac troponin I (cTnI), a key marker of myocardial injury [169]. The photoactive substrate consisted of gold nanoparticle-decorated TiO_2_ nanotube arrays (Au/TiO_2_ NTAs) coated with a carboxymethylated dextran (CM-dextran) hydrogel. The hydrogel enabled stable antibody immobilization while resisting nonspecific adsorption. Upon target binding, the formed immunocomplex impeded hole transfer from the TiO_2_ to the electron donor, causing a reduction in the photocurrent. The sensor offered a dynamic range from 0.22 pM to 2.2 nM and a detection limit of 0.1 pM (2.2 pg∙mL^−1^), outperforming standard ELISA methods. It showed good reusability, stable response over multiple cycles, and high correlation with clinical ELISA assays, enabling detection of cTnI levels below conventional thresholds.

To further expand functional versatility, recent efforts have integrated PEC with dual-mode readouts, such as colorimetry, for multimodal biomarker detection. Wang et al. developed a dual-mode PEC biosensor for ultrasensitive detection of amyloid-β peptide (Aβ_1–42_) [119], a critical biomarker of Alzheimer’s disease. A Ti_3_C_4_@Bi_2_WO_6_ Schottky heterojunction photoelectrode enhanced light harvesting and carrier separation. The immunoassay incorporated CaCO_3_@CuO_2_ nanocomposites conjugated with anti-Aβ_1–42_ antibodies and glucose oxidase (GOx). Upon Aβ binding, GOx catalyzed glucose oxidation, releasing H_2_O_2_ and gluconic acid, which degraded the CaCO_3_@CuO_2_ carriers, freeing Ca^2+^ and Cu^2+^. The Cu^2+^ ions initiated a Fenton-like reaction for colorimetric signal output, while Ca^2+^ cross-linked with alginate on the photoelectrode to form a hydrogel film that attenuated the PEC signal. This dual-mode detection yielded a linear range from 0.1 pg∙mL^−1^ to 100 ng∙mL^−1^ and a detection limit of 0.06 pg∙mL^−1^. The platform performed well in artificial cerebrospinal fluid, supporting its potential for early diagnosis of Alzheimer’s disease. Alternatively, supramolecular hydrogel systems have been employed to achieve the analyte-triggered in situ modulation of PEC responses, offering smart responsiveness. Wei et al. reported a supramolecular hydrogel-modulated PEC immunosensor for detecting human epidermal growth factor receptor 2 (HER2) (Figure 9B) [131], an important cancer biomarker. A WO_3_/SnIn_4_S_8_ heterojunction served as the photoactive layer, while an AMP-based supramolecular hydrogel doped with MnO_2_ was formed in situ upon HER2 recognition. The MnO_2_ not only absorbed incident light but blocked ion transport, significantly suppressing charge separation and shifting the PEC system into a “signal-off” mode. This smart interface yielded a broad linear range (0.1 pg∙mL^−1^ to 50 ng∙mL^−1^) and an ultralow detection limit of 0.037 pg∙mL^−1^. The system showed excellent selectivity and reproducibility in serum samples, with recovery rates from 94.3% to 105.6% and low RSDs (2.4–4.3%). This study demonstrated how supramolecular hydrogel engineering can improve PEC sensitivity and specificity for low-abundance biomarkers in clinical settings.

#### 3.3.3. Enzymes

Enzymes are highly specific biological macromolecules composed of amino acids, folded into unique three-dimensional conformations that define their catalytic activity. They facilitate a vast range of biochemical reactions by lowering activation energies and are crucial in metabolism, signal transduction, and disease progression. Given their functional significance, abnormalities in enzyme activity often correlate with pathological conditions, such as cardiovascular disorders, cancer, and inflammation. Consequently, the accurate and sensitive detection of enzymatic biomarkers has become a priority in biomedical diagnostics and therapeutic monitoring [170,171,172,173]. PEC sensors, when coupled with hydrogels, offer an innovative platform for enzyme detection, enabling improved specificity, tunable signal amplification, and compatibility with real biological samples.

Wang et al. developed a label-free PEC aptasensor for thrombin detection using a signal-amplifying hybrid of silver and TiO_2_ nanoparticles embedded within a three-dimensional nitrogen-doped graphene hydrogel (3DNGH) [122]. The photoactive Ag/TiO_2_/3DNGH material was synthesized via a one-step hydrothermal method, combining high surface area, improved conductivity, and localized surface plasmon resonance (LSPR) effects to boost the photocurrent. The thrombin-specific aptamer was immobilized onto the Ag/TiO_2_/3DNGH-modified ITO electrode via Ag–S bonding. Upon target binding, thrombin hindered interfacial charge transfer, leading to a decrease in the photocurrent. The PEC aptasensor achieved a broad linear range from 0.01 pM to 10 μM and an exceptionally low detection limit of 3 fM. The hydrogel not only offered a scaffold for nanoparticle dispersion and biomolecule immobilization but facilitated efficient charge transport. The sensor displayed excellent selectivity and stability, and its practical applicability was demonstrated through successful recovery in spiked samples, underscoring its potential in protein diagnostics.

**Figure 10:**
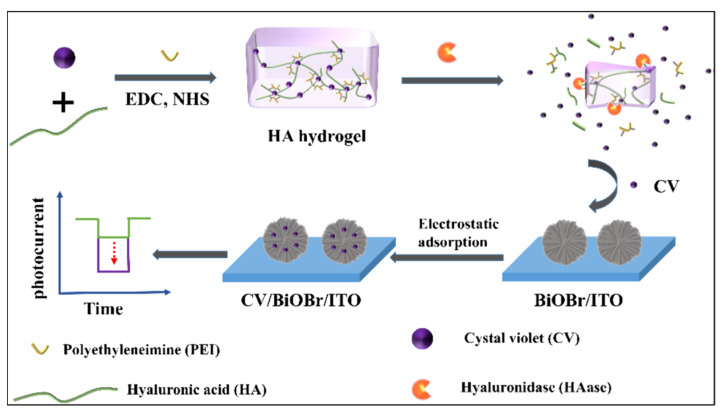
Schematic illustration of a “signal-on” photoelectrochemical (PEC) biosensor for hyaluronidase (HAase) detection. Enzymatic degradation of the HA-based hydrogel by HAase releases cationic dye crystal violet (CV), which enhances visible light absorption and charge separation on BiOBr, resulting in increased cathodic photocurrent. Reproduced with permission from reference [110].

While this approach relies on hydrogel-assisted LSPR signal amplification, enzyme-triggered degradation mechanisms can also be harnessed to enable self-actuated sensing. Hu et al. introduced a novel “signal-on” PEC biosensing platform for detecting hyaluronidase (HAase) activity using an enzyme-responsive hydrogel and BiOBr nanoflowers (Figure 10) [110]. The strategy relied on a hyaluronic acid (HA)-based hydrogel cross-linked with polyethyleneimine (PEI), which encapsulated the photosensitizing dye crystal violet (CV). Upon exposure to HAase, the hydrogel underwent enzymatic degradation, releasing CV molecules. The liberated CV adsorbed onto BiOBr nanoflowers on an ITO electrode, enhancing visible light absorption and electron–hole separation, thus boosting the cathodic photocurrent. This system enabled a “signal-on” response mechanism. The PEC sensor showed a logarithmic response over a broad HAase activity range (0.10–120 U/mL), with an ultralow detection limit of 0.034 U/mL. Importantly, the hydrogel functioned as a gatekeeper for signal molecules and offered selective release based on enzymatic activity. The platform was successfully applied to human urine samples, achieving high recoveries and agreement with ELISA results. This work exemplifies a simple, cost-effective, and environmentally friendly hydrogel-mediated PEC assay for enzyme activity evaluation in clinical diagnostics.

### 3.4. Other Applications

In addition to the detection of conventional molecular targets, hydrogel-assisted PEC sensing platforms have been increasingly extended to a broader range of application areas, including pathogen monitoring, optoelectronic detection, and flexible neurointerfaces. This section presents several representative examples, highlighting the broad potential of hydrogels in facilitating functional integration and driving the development of intelligent PEC devices.

#### 3.4.1. Detection of *Escherichia coli*

*Escherichia coli* (*E. coli*) is a widely studied pathogenic microorganism associated with waterborne and foodborne illnesses. The accurate and rapid detection of *E. coli* is crucial for public health and food safety. Hao and Wang et al. developed a sensitive ratiometric PEC aptasensor based on potentiometric resolution for the detection of *E. coli* [126]. The sensor utilizes two non-metallic photoactive materials immobilized onto different regions of a single ITO electrode: carbon dot-loaded three-dimensional graphene hydrogel (C-dots/3DGH), serving as the cathodic component, and graphitic carbon nitride (g-C_3_N_4_), as the anodic counterpart. The two materials operate at distinct bias voltages (−0.4 V for C-dots/3DGH and +0.15 V for g-C_3_N_4_), enabling clearly separated and interference-free photoresponses. Aptamers specific to *E. coli* were anchored to the C-dots/3DGH surface. Upon binding of *E. coli*, steric and electrostatic hindrance reduced the cathodic photocurrent while the anodic signal from g-C_3_N_4_ remained stable, allowing for ratiometric signal readout via the I_c_/I_a_ ratio. The graphene-based hydrogel played a dual role, both enhancing charge transport and providing a porous scaffold for aptamer immobilization. The sensor demonstrated excellent analytical performance, including a broad linear detection range from 2.9 to 2.9 × 10^6^ cfu∙mL^−1^ and a remarkably low detection limit of 0.66 cfu∙mL^−1^. Moreover, it exhibited high reproducibility, operational stability, and tolerance to variable light intensity and ionic strength. When applied to real milk samples, the sensor achieved recoveries between 98.6% and 102%, underscoring its practical applicability in food safety and environmental monitoring.

#### 3.4.2. Photodetectors

Hydrogel-integrated PEC photodetectors have emerged as promising platforms for wearable, self-powered optoelectronic systems due to their intrinsic softness, ion-conducting nature, and mechanical resilience. Recent advances have demonstrated their utility across a range of designs, materials, and signal conversion strategies, enabling functionalities such as ultraviolet detection, broadband response, and even intelligent imaging.

**Figure 11:**
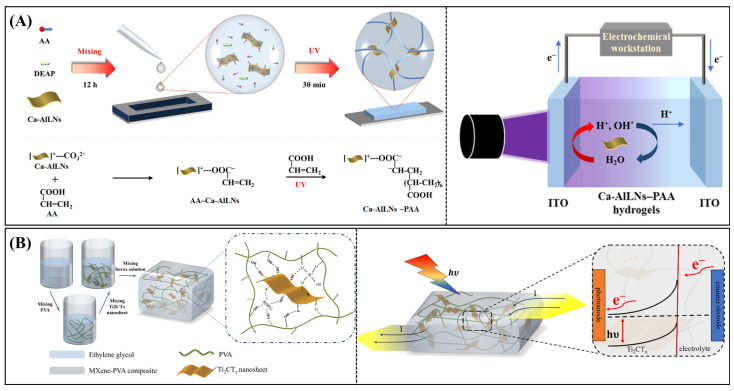
(**A**) Schematic illustration of a multifunctional hydrogel composed of polyacrylic acid (PAA) reinforced with calcium–aluminum layered double hydroxide (Ca–Al LDH) nanosheets, enabling integrated sensing of mechanical strain, temperature, and ultraviolet (UV) radiation. Reproduced with permission from reference [174]. (**B**) Illustration of a stretchable and self-healing PEC photodetector constructed using a borate-crosslinked polyvinyl alcohol (PVA) hydrogel embedded with Ti_2_CT_x_ (MXene) nanosheets. Reproduced with permission from reference [116].

Feng et al. developed a multifunctional photo-crosslinked hydrogel composed of polyacrylic acid (PAA) and Ca–Al layered double hydroxide (LDH) nanosheets for integrated sensing of mechanical force, temperature, and ultraviolet (UV) radiation (Figure 11A) [174]. The LDH reinforcement imparted the hydrogel with enhanced mechanical strength, UV resistance, and robust adhesion. Under UV illumination (≤405 nm), the hydrogel generated protons and electrons, producing a measurable photocurrent for self-powered detection. Simultaneously, resistance variations enabled strain sensing, and ion diffusion facilitated thermal signal transduction via a Seebeck-type mechanism. The sensor exhibited an excellent photoresponse at 365 nm, including a photocurrent density of 186 nA∙cm^−2^, a responsivity of 1.58 μA∙W^−1^, and a detectivity of 1.33 × 10^7^ Jones. This design exemplifies a rare instance of a hydrogel-only device achieving simultaneous mechanical, thermal, and optical sensing, suggesting great promise for human–machine interfaces and wearable electronics. To further improve spectral response and mechanical resilience, other groups have explored hydrogel–electrolyte integration with broadband semiconductor materials. Sha et al. constructed a broadband, self-powered PEC photodetector using ultrathin Bi_2_O_2_Se nanosheets as the photoanode and a cellulose/carboxymethyl cellulose (CMC) hydrogel as the electrolyte [109]. The Bi_2_O_2_Se material, synthesized via a mild aqueous process, provided high photoactivity from 365 to 850 nm, while the cellulose-based hydrogel offered mechanical flexibility and ionic conductivity. Under illumination, photogenerated carriers were efficiently separated and extracted, with OH^−^ in the hydrogel facilitating hole scavenging. The device demonstrated a responsivity of 0.68 mA∙W^−1^ and a detectivity of 2.44 × 10^8^ Jones at 365 nm, alongside a rapid response time (85/103 ms). Importantly, the sensor retained over 70% of its output after 100 mechanical deformations, validating its durability for real-world use.

In addition to mechanical flexibility, attributes such as self-healing and robustness are also being incorporated into photodetector designs through hydrogel engineering. Bao and Qi et al. introduced a stretchable and self-healing PEC photodetector built upon a polyvinyl alcohol (PVA) hydrogel embedded with Ti_2_CT_x_ (MXene) nanosheets, crosslinked using borate ions (Figure 11B) [116]. The MXene enhanced the photocarrier mobility and light responsiveness, while the hydrogel matrix provided resilience and reusability. The photodetector achieved a photocurrent density of 0.333 μA∙cm^−2^ and a responsivity of 2.96 μA∙W^−1^ under 0.3–0.6 V bias and illumination intensities between 60 and 150 mW∙cm^−2^. It maintained over 70% of its electrical output after 1500 mechanical cycles and preserved 73% performance after 50 autonomous healing events. This system exemplifies how combining soft electronics and conductive hydrogels can create sustainable, adaptable optoelectronic devices. Lin et al. demonstrated a hydrogel-enabled, self-powered UV photodetector that harnesses water molecule polarization within a conductive Gr–PEDOT/Alg(Fe^3+^)–TiO_2_ architecture [127]. TiO_2_ was deposited on a PET substrate, while the intermediate PEDOT-doped ferric alginate hydrogel provided ionic conductivity and polarization functionality. Upon 360 nm light exposure, internal electric fields aligned free water molecules, generating transient photocurrents without external bias. The photodetector exhibited a responsivity of 4.32 mA∙W^−1^ and a detectivity of 9.09 × 10^9^ Jones, with response times of 45.6 ms (rise) and 34.5 ms (fall). An 8 × 8 sensor array demonstrated image recognition capabilities, achieving 99% accuracy when coupled with a convolutional neural network, even under bending or oblique lighting. This system highlights the potential of hydrogel photophysics for smart sensing and integrated optoelectronic-AI platforms.

#### 3.4.3. Flexible Bioelectronic Interfaces

Flexible bioelectronic interfaces aim to bridge biological systems and artificial electronic devices by enabling seamless, conformal, and dynamic integration for physiological signal detection, neuromorphic computing, and real-time in vivo monitoring [175,176,177]. These systems must balance mechanical compliance with high-fidelity electronic transduction, biocompatibility, and stability under physiological conditions. Hydrogels, with their tunable mechanics, high water content, and tissue-like softness, have emerged as ideal interface materials, especially when integrated with PEC or optoelectronic transduction elements.

Zhao et al. developed a high-efficiency semi-artificial photoelectrochemical (PEC) bio-photocathode that harnesses Photosystem I (PSI) as the primary light-harvesting complex [178]. PSI was immobilized within a redox hydrogel matrix composed of poly(vinyl)imidazole Os(bipy)_2_Cl and polyethylene glycol diglycidyl ether (PEGDGE), which was further nanostructured using single-walled carbon nanotubes (SWCNTs) covalently integrated into the network. This configuration formed a conductive and hydrated scaffold that not only stabilized PSI but facilitated efficient electron transfer from PSI to the electrode. Upon red light illumination, photoexcited electrons generated by PSI were shuttled through the Os-based redox mediator to the SWCNTs and subsequently to the electrode, resulting in a cathodic photocurrent. Under oxygen-saturated conditions, the system delivered a remarkable steady-state photocurrent density exceeding 2.0 mA∙cm^−2^—setting a new benchmark for semiconductor-free PSI-based devices. Furthermore, an incident photon-to-current efficiency (IPCE) of 10% was achieved at a low illumination intensity of 0.14 mW∙cm^−2^. The incorporation of SWCNTs markedly improved charge transport dynamics and hydrogel stability, underscoring the platform’s potential for sustainable, protein-based solar energy conversion systems.

**Figure 12:**
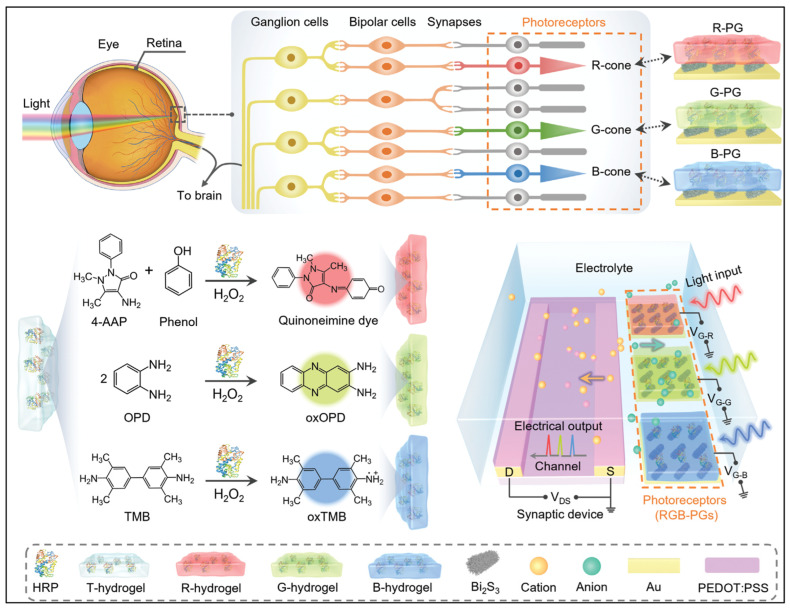
Schematic illustration of a biomimetic photoelectrochemical synapse that emulates retinal light perception and synaptic behavior by integrating enzyme-responsive chromogenic hydrogels with a Bi_2_S_3_-based photoelectrode within an organic photoelectrochemical transistor (OPECT) architecture. Reproduced with permission from reference [69].

Beyond energy applications, hydrogel-enabled biointerfaces are also being applied in neuromorphic systems that emulate biological signal transduction. Jiang, Lin, and Zhao et al. introduced a biomimetic photoelectrochemical synapse that mimics the functional attributes of the human retina by integrating enzyme-responsive colored hydrogels with a Bi_2_S_3_-based photoelectrode in an OPECT configuration (Figure 12) [69]. Three distinct chromogenic hydrogels—red, green, and blue—were prepared by horseradish peroxidase (HRP)-mediated oxidation reactions in gelatin matrices, each tailored to absorb light in specific visible regions. These hydrogels served as dynamic photogating elements in conjunction with a MOF-derived Bi_2_S_3_ photoelectrode, regulating ionic migration and thereby modulating the channel current in a PEDOT/PSS layer without the need for external gate voltage. Beyond color-selective photodetection, the device exhibited neuromorphic capabilities, including short- and long-term synaptic plasticity. The presence of hydrogen peroxide modulated hydrogel coloration enabled chemically gated memory transitions, such as paired-pulse facilitation (PPF) and forgetfulness. Additionally, the hydrogel’s color could be reversibly tuned using ascorbic acid, mimicking photopigment regeneration in cone cells. A 4 × 4 synaptic array was fabricated to perform RGB image recognition and logic operations using wavelength and H_2_O_2_ dual-encoded inputs. This work establishes a novel platform for integrating chemical, optical, and electrical signaling in bioinspired neuromorphic systems, paving the way for next-generation artificial retinas and soft neurocomputing interfaces.

## 4. Conclusions and Perspectives

In recent years, the integration of hydrogels into PEC sensors has rapidly emerged as an innovative strategy, significantly enhancing the performance, versatility, and applicability of PEC sensing platforms. The intrinsic attributes of hydrogels—including exceptional biocompatibility, tunable mechanical properties, high hydration levels, and structural versatility—have endowed PEC sensors with unprecedented functionalities. These hydrogel-enhanced PEC platforms have been successfully employed in diverse applications ranging from environmental monitoring, food safety analysis, and clinical diagnostics, to wearable and flexible bioelectronics (Table 1). Hydrogels in PEC sensors generally function via multiple pathways: providing structural scaffolding and mechanical flexibility, enhancing charge transfer efficiency through conductive matrices, enabling stimuli-responsive release mechanisms for signal amplification, and conferring antifouling characteristics that improve long-term stability. Notably, hydrogels facilitate the integration of recognition elements, such as nucleic acids, enzymes, and aptamers, within the PEC architecture, greatly broadening the scope of detectable analytes from simple ions and small organic molecules to complex biomacromolecules, like proteins and microRNAs.

However, the performance advantages claimed by hydrogel integration remain difficult to quantitatively generalize, as few studies offer side-by-side comparisons with non-hydrogel PEC systems under identical conditions. Metrics such as sensitivity, detection limits, and stability are often influenced by coexisting factors like photoactive materials, nanostructures, or signal transduction strategies. Therefore, future research should emphasize controlled comparisons and standardized evaluation protocols to clarify the specific roles and benefits of hydrogels.

Moreover, while hydrogels can facilitate molecular recognition and antifouling properties, they may also introduce practical complications. Fabrication methods—such as DNA-hydrogel self-assembly or enzyme-immobilized network formation—can be time-consuming, cost-prohibitive, or difficult to scale. For instance, stimuli-responsive DNA hydrogels typically require multi-step enzymatic ligation and careful sequence design, which may limit industrial feasibility. These issues are rarely addressed in the literature, yet they are critical for real-world deployment.

Stability also remains an underexplored challenge. Hydrogel swelling, dehydration, and degradation due to enzymatic or oxidative conditions can lead to signal drift or sensor failure, particularly in long-term or in vivo applications. While antifouling behavior is often cited as an advantage, the actual durability of hydrogel coatings under continuous exposure to biological fluids or environmental contaminants is seldom quantified. Parameters such as mechanical fatigue, ionic permeability, and signal baseline shift warrant deeper investigation.

Furthermore, although hydrogels have been integrated with a variety of traditional semiconductors, the interface between hydrogels and advanced photoactive materials—such as covalent organic frameworks (COFs), single-atom catalysts (SACs), or perovskites—remains poorly understood. Specific interfacial challenges include lattice mismatch, unfavorable band alignment, and inefficient charge carrier transfer. Addressing these issues will require new coupling strategies (e.g., interfacial linkers, redox mediators, or hybrid gels) and in situ spectroscopic characterization to reveal underlying mechanisms.

Despite remarkable progress, several critical challenges and opportunities remain to be addressed.

First, further advancements in hydrogel design are needed to enhance long-term stability and durability under physiological and environmental conditions. Achieving precise control over the mechanical properties and degradation kinetics of hydrogels remains vital, particularly for wearable or implantable PEC systems. This includes tailoring hydrogels for resistance to enzymatic degradation, dehydration, or fouling over extended use in biological environments.

Second, current PEC-hydrogel sensors often rely on complex and multi-step fabrication procedures. Streamlined methodologies and large-scale fabrication strategies must be developed to improve practical feasibility, scalability, and reproducibility, enabling commercial translation of these innovative platforms. Emerging approaches, such as hydrogel microprinting, 3D bioprinting, and UV-curable formulations, offer promise for rapid prototyping and batch production.

Third, while substantial progress has been made in integrating hydrogels with traditional inorganic photoactive materials, the incorporation of emerging materials, such as two-dimensional nanomaterials, single-atom catalysts, and covalent organic frameworks (COFs), into hydrogel matrices remains largely unexplored. Tuning interfacial energetics—such as Fermi level alignment and interfacial dipole orientation—within hydrogel–semiconductor composites represents a promising direction for enhancing photocarrier separation and PEC efficiency.

Fourth, miniaturization and portability are essential considerations for future PEC hydrogel sensors intended for field-deployable diagnostics and continuous real-time monitoring. Integrating hydrogel-based PEC sensors with microfluidic devices, and wearable electronics could open new avenues for personalized healthcare and environmental monitoring. Additionally, exploring dual- or multi-modal detection strategies, such as coupling PEC transduction with colorimetric, electrochromic, or fluorescence signals, could significantly enhance detection reliability and enable visual on-site readouts. Furthermore, adaptive hydrogels capable of mechanical actuation or autonomous feedback in closed-loop systems may support intelligent biosensing applications.

Finally, bio-inspired PEC systems represent an exciting frontier, particularly regarding flexible bioelectronic interfaces and neuromorphic devices. Mimicking biological processes, such as synaptic plasticity, memory formation, and adaptive perception, through hydrogel-based PEC devices offers profound potential for artificial intelligence interfaces, neural prosthetics, and advanced sensory networks. Nevertheless, a deeper mechanistic understanding of ion diffusion dynamics, recombination kinetics, and electric double-layer modulation at the hydrogel–electrode–biointerface is critical to unlocking the full potential of these systems.

In summary, hydrogel-promoted PEC sensors are transforming the analytical landscape, delivering innovative solutions across biomedical, environmental, and technological fields. Continued interdisciplinary collaboration among chemists, material scientists, engineers, and biomedical researchers will undoubtedly accelerate advancements, driving the translation of these pioneering platforms from laboratory proof-of-concepts toward robust, commercial technologies with real-world impact.

## Figures and Tables

**Table 1:** Summary of representative hydrogel-based photoelectrochemical (PEC) sensors.

Targets	Hydrogel	Functions of Hydrogel	Photoactive Materials	Dynamic Range	LOD	Applications	Ref.
Cu^2+^	PVA hydrogel	Antifouling barrier	PI film/LIG-In-CdS/Hydrogel	1.28 ng∙mL^−1^–5.12 μg∙mL^−1^	--	Sweat sample	[60]
Ru^3+^	PAAm hydrogel	Confined reactor for 2D COF synthesis	ITO/COF(TTA-DHTA)	0.3–30 μM	--	--	[133]
Dopamine	Acrylamide-based antibiofouling hydrogel	Antifouling interface	Ti/TiO_2_ NTPCs/Z-MOF/Hydrogel	10–500 nM	6.0 nM	--	[118]
Dopamine	PEDOT/PSS/PR-PEGMA/AAm	Biocompatible and flexible interface	Hydrogel/AgInS_2_	0.2–4 μM	64 nM	In vivo monitoring	[107]
Glucose	GOx & HRP DNA hydrogel	Conductive scaffold	FTO/In_2_O_3_–CdS	10–500 nM	9.62 nM	--	[113]
H_2_O_2_, Glucose	Three-dimensional nitrogen-doped graphene hydrogel (3DNGH)	Biocompatible enzyme matrix	ITO/3DNGH@ZnO/HRP	H_2_O_2_: 0.001–5 mM Glucose: 0.002–8 mM	H_2_O_2_: 0.33 μM Glucose: 0.66 μM	Orange juice	[141]
Glucose, Sarcosine, Lactate	A Fc–PEG–SS–gelatin hydrogel (amide bond-based)	Conductive 3D matrix	Silicon/Fc-containing hydrogel	Glucose: 0.5–2.5 mM; Sarcosine: 0.3–1.5 mM; Lactate: 1.0–3.0 mM	Glucose: 179 μM; Sarcosine: 16 μM; Lactate: 780 μM	Sweat sample	[128]
Guanine	Polyaniline hydrogel (PAniHs)	Self-redox conductive hydrogel matrix	GCE/PAniHs/PTB7–Th/XOD	0.1–80 μM	0.02 μM	Aciclovir tablets	[117]
Hydrogen	Viologen-modified polyethyleneimine	Redox-active enzyme matrix	[NiFe]-H_2_ase/PS1–Pt	1.6–24 µM	0.81 µM	--	[142]
Tetracycline	Three-dimensional nitrogen-doped graphene hydrogel (3DNGH)	Conductive 3D scaffold	ITO/3DNGH/BiPO_4_/Apt	0.1 nM–1 μM	0.033 nM	Milk sample	[124]
Chloramphenicol (CAP)	Three-dimensional nitrogen-doped graphene hydrogel (NGH)	Conductive matrix for p–n heterojunction	ITO/NGH/MoS_2_-Apt	32.3 ng∙L^−1^–96.9 μg∙L^−1^	3.23 ng∙L^−1^	Honey sample	[125]
Chlorpyrifos	3D nitrogen-doped graphene hydrogel (NGH)	Plasmonic conductive matrix	ITO/TiO_2-x_	0.05 ng∙mL^−1^–0.5 µg∙mL^−1^	0.017 ng∙mL^−1^	Water sample	[121]
Pentachlorophenol (PCP)	PEGDA hydrogel coated with calcium alginate (CA)	Freestanding optical fiber matrix	PEGDA hydrogel fiber(Au@Ag NWs and PCN-224(Zn)@TiO_2_)/CA	PEC: 0.05–1000 ng∙mL^−1^; FL: 0.01 pg∙mL^−1^–1 μg∙mL^−1^	PEC: 2.9 fg∙mL^−1^; FL: 0.11 fg∙mL^−1^	In vivo monitoring	[150]
Atrazine(ATZ), Acetamiprid(ACP), Carbendazim (CBZ)	MeHA–HA Hydrogel	Swellable microneedle array	ITO/Bi2S3-Bi2O3/Au NPs-Apt/MeHA–HA Hydrogel	ATZ: 0.1 fg∙mL^−1^–0.1 ng∙mL^−1^	ATZ: 0.029 fg∙mL^−1^; ACP: 5.5 fg∙mL^−1^; CBZ: 21 fg∙mL^−1^	In vivo detection (mouse and leaves)	[129]
Ochratoxin A	Three-dimensional graphene hydrogel (3DGH)	Conductive scaffold	ITO/Co, N-co-doped TiO_2_@3DGH/PB	1–500 ng∙mL^−1^	0.29 ng∙mL^−1^	Corn juice	[132]
Ochratoxin A	Polyaniline hydrogel	In situ conductive PANI matrix for signal amplification	ITO/GO-MoS_2_-CdS/PAMAM	0.0001–0.1 ng∙mL^−1^	0.05 pg∙mL^−1^	Red wine	[112]
Microcystin-LR	Three-dimensional nitrogen-doped graphene hydrogel (NGH)	Conductive 3D NGH scaffold	ITO/Fe_2_O_3_/NGH/Apt	1 pM–5 nM	0.23 pM	Water sample	[120]
4-Nitrophenol	3D Graphene Hydrogel (GH)	3D conductive scaffold	ITO/Bi_2_WO_6_@GH/PPy	5.0 × 10^−12^–1.0 × 10^−7^ M	5.78 × 10^−13^ M	Detection in PM2.5	[123]
microRNA-141, microRNA-21	3D Graphene Hydrogel (3DGH)	3D conductive scaffold	ITO/CdTe-3DGH	miRNA-141: 1.0–10^4^ fM; miRNA-21: 1.0–10^5^ fM	miRNA-141: 0.63 fM; miRNA-21: 0.29 fM	Serum sample	[163]
microRNA-155	HA–PEI–DNA@TiO_2_ NPs hydrogel	Target-responsive DNA hydrogel for signal amplification	FTO/TiO_2_ NPs	1.0 fM–100 pM	0.41 fM	Cell lysates	[114]
microRNA-21	DNA hydrogel	Stimuli-responsive DNA hydrogel for signal amplification	ITO/g-C_3_N_4_ & CdS	10 aM–1 nM	3.2 aM	Cell Lysates	[111]
HIgG	SA/GO-Ca^2+^ hydrogel	Ca^2+^-induced hydrogel gelation for signal modulation	ITO/CdS	100 fg∙mL^−1^–100 ng∙mL^−1^	50 fg∙mL^−1^	Serum sample	[130]
HIgG	Pt nanocube-embedded gelatin hydrogel (PGH)	Color-gated PEC signal modulation	FTO/CdIn_2_S_4_	10 fg∙mL^−1^–10 ng∙mL^−1^	10 fg∙mL^−1^	Serum sample	[108]
CEA	Polymerized ionic liquid hydrogel	Imprinted recognition matrix and ion-conductive layer	GCE/MoSe_2_/HGNBs	0.05–5.0 ng∙mL^−1^	11.2 pg∙mL^−1^	Serum sample	[115]
cTnI	Carboxymethylated dextran	Stable support	Ti/TiO_2_ NTA/Au NPs	0.22 pM–2.2 nM	0.1 pM (2.2 pg/mL)	Human serum	[169]
Amyloid-β peptide (Aβ_1-42_)	Calcium alginate hydrogel	In Situ formation for signal-off regulation	Ti_3_C_2_@Bi_2_WO_6_	0.1 pg∙mL^−1^–100 ng∙mL^−1^	0.06 pg∙mL^−1^	Artificial cerebrospinal fluid	[119]
HER2	MnO_2-_doped/AMP supramolecular hydrogel	In Situ formation for signal-off regulation	ITO/WO_3_/SnIn_4_S_8_	0.1 pg∙mL^−1^–50 ng∙mL^−1^	0.037 pg∙mL^−1^	Human serum	[131]
Thrombin	Ag/TiO_2_/3D nitrogen doped graphene hydrogel (3DNGH)	Conductive scaffold	ITO/Ag/TiO_2_/3DNGH-Apt	0.01 pM–10 pM	3 fM	Serum sample	[122]
Hyaluronidase	HA–PEI hydrogel	Target-responsive hydrogel for signal amplification	ITO/BiOBr & (crystal violet)	0.10–120 U∙mL^−1^	0.034 U∙mL^−1^	Urine sample	[110]
*Escherichia coli*	3D graphene hydrogel loaded with carbon dots (C-dots/3DGH)	Conductive scaffold	ITO/C-dots/3DGH-Apt	2.9–2.9 × 10^6^ cfu∙mL^−1^	0.66 cfu∙mL^−1^	Milk sample	[126]
Mechanical force, temperature, UV light	Ca-AlLNs–PAA	Multi-responsive	PEDOT/PSS/ZnO/agarose/PVA Ca-AlLNs–PAA	Temperature: 0–78 °C; UV: ≤405 nm	1.33 × 10^7^ Jones	Skin sensor	[174]
Photodetector	cellulose/CMC	Ionic conductivity and mechanical flexibility	PET/ITO/Bi_2_O_2_Se	365–850 nm; 6.84–36.16 mW∙cm^−2^	2.44 × 10^8^ Jones	Rotational speed measurement	[109]
Photodetector	PVA/Ti_2_CT_x_ MXene	Electron-conductive matrix	PVA/Ti_2_CT_x_ MXene	Light intensity: 60–150 mW∙cm^−2^	--	Wearable electronics	[116]
Photodetector	PEDOT/Alg(Fe^3+^) hydrogel	Electrochromic and conductive scaffold	PET/TiO_2_/hydrogel/Gr	225–505 nm, 0–660 μW∙cm^−2^	9.09 × 10^9^ Jones	Image acquisition	[127]
Photosystem I	(poly(vinyl)imidazole/Os(bipy)_2_Cl)/PEGDGE	Conductive scaffold	Au/SWCNTs/PSI-POs	Light intensity: 0.14–471 mW∙cm^−2^	--	solar-to-electricity conversion	[178]
Color-specific light signals	Gelatin hydrogels	As chromogenic reaction matrix	Au/Bi_2_S_3_	Qualitative RGB color range, biomolecule concentration-dependent PSC modulation	Response to H_2_O_2_ down to 0.1 mM	Artificial visual synapse with RGB image	[69]

## Data Availability

Not applicable.

## Abbreviations

The following abbreviations are used in this manuscript:

4NP	4-Nitrophenol
ACP	Acetamiprid
Alg	Alginate
ATZ	Atrazine
CBZ	Carbendazim
C-dots	Carbon dots
CMC	Carboxymethyl cellulose
CEA	Carcinoembryonic antigen
cTnI	Cardiac troponin I
CLIA	Chemiluminescent immunoassay
CAP	Chloramphenicol
cDNA	Complementary DNA
CV	Crystal violet
DBCO	Dibenzocyclooctyne
*E. coli*	*Escherichia coli*
Fc	Ferrocene
GCE	Glassy carbon electrode
GOx	Glucose oxidase
AuNPs	Gold nanoparticles
GH	Graphene hydrogel
g-C_3_N_4_	Graphitic carbon nitride
HRP	Horseradish peroxidase
HER2	Human epidermal growth factor receptor 2
HIgG	Human immunoglobulin G
HA	Hyaluronic acid
HAase	Hyaluronidase
H_2_ase	Hydrogenase
IPCE	Incident photon-to-current efficiency
ITO	Indium tin oxide
LDH	Layered double hydroxide
LOD	Limit of detection
LSPR	Localized surface plasmon resonance
MIL-125	Materials of Institute Lavoisier-125, a titanium-based metal–organic framework.
MC-LR	Microcystin-LR
miRNA	MicroRNA
MIPs	Molecularly imprinted polymers
NGH	Nitrogen-doped graphene hydrogel
OTA	Ochratoxin A
OPD	*o*-Phenylenediamine
OPECT	Organic photoelectrochemical transistor
PPF	Paired-pulse facilitation
PCP	Pentachlorophenol
PEC	Photoelectrochemical
PSI	Photosystem I
PGH	Platinum nanocube-embedded gelatin hydrogel
PEDOT	Poly(3,4-ethylenedioxythiophene)
PAMAM	Poly(amidoamine)
PEGMA	Poly(ethylene glycol) methacrylate
PTB7-Th	Poly[[2,6′-4,8-di(5-ethylhexylthienyl) benzo[1,2-b;3,3-b] dithiophene][3-fluoro-2-(2-ethylhexy) carbonyl-thieno[3,4-b]thiophenediyl]]
PAM	Polyacrylamide
PAA	Polyacrylic acid
PANI	Polyaniline
PEG	Polyethylene glycol
PEGDA	Polyethylene glycol diacrylate
PEGDGE	Polyethylene glycol diglycidyl ether
PEI	Polyethyleneimine
PVA	Polyvinyl alcohol
RSD	Relative standard deviation
SWCNTs	Single-walled carbon nanotubes
SA	Sodium alginate
Tc	Tetracycline
Ti_2_CT_x_	Transition metal carbides and nitrides (Ti_2_CT_x_)
UV	Ultraviolet
XOD	Xanthine oxidase
Z-MOFs	Zwitterionic metal–organic frameworks
